# Structural intermediates in the low pH-induced transition of influenza hemagglutinin

**DOI:** 10.1371/journal.ppat.1009062

**Published:** 2020-11-30

**Authors:** Jingjing Gao, Miao Gui, Ye Xiang

**Affiliations:** Beijing Advanced Innovation Center for Structural Biology, Beijing Frontier Research Center for Biological Structure, Center for Infectious Disease Research, Department of Basic Medical Sciences, School of Medicine, Tsinghua University, Beijing, China; Institut Pasteur, FRANCE

## Abstract

The hemagglutinin (HA) glycoproteins of influenza viruses play a key role in binding host cell receptors and in mediating virus-host cell membrane fusion during virus infection. Upon virus entry, HA is triggered by low pH and undergoes large structural rearrangements from a prefusion state to a postfusion state. While structures of prefusion state and postfusion state of HA have been reported, the intermediate structures remain elusive. Here, we report two distinct low pH intermediate conformations of the influenza virus HA using cryo-electron microscopy (cryo-EM). Our results show that a decrease in pH from 7.8 to 5.2 triggers the release of fusion peptides from the binding pockets and then causes a dramatic conformational change in the central helices, in which the membrane-proximal ends of the central helices unwind to an extended form. Accompanying the conformational changes of the central helices, the stem region of the HA undergoes an anticlockwise rotation of 9.5 degrees and a shift of 15 Å. The HA head, after being stabilized by an antibody, remains unchanged compared to the neutral pH state. Thus, the conformational change of the HA stem region observed in our research is likely to be independent of the HA head. These results provide new insights into the structural transition of HA during virus entry.

## Author summary

Influenza virus is a threat to human health which can cause seasonal and pandemic flu. As the most important antigen of influenza virus, hemagglutinin (HA) is a primary target of drugs and vaccines. However, a universal vaccine is still lacking and drug resistance is an emerging problem due to the fast mutation and evolution of influenza virus. HA is a transmembrane viral surface protein which is responsible for interacting with host cell receptors and mediating virus entry. In endosomes of the host cells, HA is triggered by low pH and undergoes a series of conformational change which is a conserved but highly dynamic process. Capturing the intermediate states has been a challenge. In this research, we used a neutralizing antibody to stabilize the HA, fast froze the low pH treated sample in vitreous ice and determined several intermediate structures of the HA by computational 3D classification. Our research revealed structural details of HA intermediates upon low pH treatment, deepening our understanding of virus entry, potentially providing new hints for universal flu therapy and vaccines.

## Introduction

The influenza virus hemagglutinin (HA) glycoprotein is synthesized as a ~560 amino acid precursor protein (HA0) of a single polypeptide chain, which is cleaved into the HA1 and HA2 subunits linked by a disulfide bond, either in the cell during virus assembly or on the cell surface during virus entry by host cell proteases[1,2]. Three HA1/HA2 heterodimers assemble to form a trimer spike protruding from the virus envelope, with the HA2 and the short N- and C-terminal fragments of HA1 forming membrane-proximal stem and the large middle fragment of HA1 forming the membrane-distal head. The head encompasses the sialic acid receptor binding domains (RBDs), while the stem has a hydrophobic fusion peptide at the N-terminus and the transmembrane helix at the C-terminus of each HA2. Influenza viruses enter the host cells through receptor-mediated endocytosis. Triggered by the decrease in pH in late endosomes during virus entry, HA undergoes conformational changes that underlie its transition from a prefusion state to a postfusion state [3,4]. Structures of the prefusion HA before or after protease cleavage and the six-helix bundle structure of the postfusion HA2 trimer provide insights into the initial and final stages of HA during virus entry [5–8], respectively. However, molecular snapshots of HA in the intermediate states of the pre-postfusion transition, which are important for the development of universal antiviral drugs and vaccines, remain to be elucidated.

In the present research, by using cryo-EM and 3D classification, we determined the structures of HA in low pH induced intermediate states. We showed that, upon low pH treatment, HA undergoes dramatic conformational change to release the fusion peptide and to splay the central helices.

## Results

### Identification of a neutralizing antibody that stabilizes the intermediate states of HA

Previous studies suggested that the pH-induced conformational change of HA involves multiple steps [9–14]. The fusion peptide and the nearby stem region of HA could undergo initial conformational changes that further prime subsequent conformational changes, disassociation of the HA1 head and structure rearrangement of the HA2 stem [13,15,16]. Based on these observations, it should be possible to trap the pre-postfusion transition of the HA trimer in possible intermediate states by stabilizing the HA1 head.

We screened neutralizing antibodies reported in the literature that target the HA1 head [17–26]. Screening was performed to identify HA-antibody Fab complexes in which the HA head could be stabilized while the stem region underwent conformational changes upon pH change. Through initial biochemistry and cryo-EM data analyses (S1 and S2 Figs), we found that F005-126, a neutralizing antibody targeting the HA head of H3N2 viruses, fulfills all our requirements. SDS-page gel analysis showed that the ecto HA-Fab complex is trypsin resistant at pH 5.2 (S1 Fig), indicating that the binding of Fab F005-126 prevents the low pH-induced conformational transition to the postfusion state. Further cryo-EM analysis of the sample showed that the HA (strain A/Hong Kong/1/1968) with the bound Fab F005-126 was mostly intact at pH 5.2 (S2 Fig). However, aggregations of particles were detected shortly after the low pH treatment through the dynamic light scattering (DLS) analysis (S3 Fig) and were also observed in the cryo-EM images (S2B Fig), suggesting possible exposure of the hydrophobic fusion peptide at the stem region while the head was still stable and the entire HA remained intact. We then determined the cryo-EM structures of the full-length HA in complex with Fab F005-126 at pH 7.8 and pH 5.2.

### Cryo-EM structure of HA-Fab complex at neutral pH

**Fig 1:**
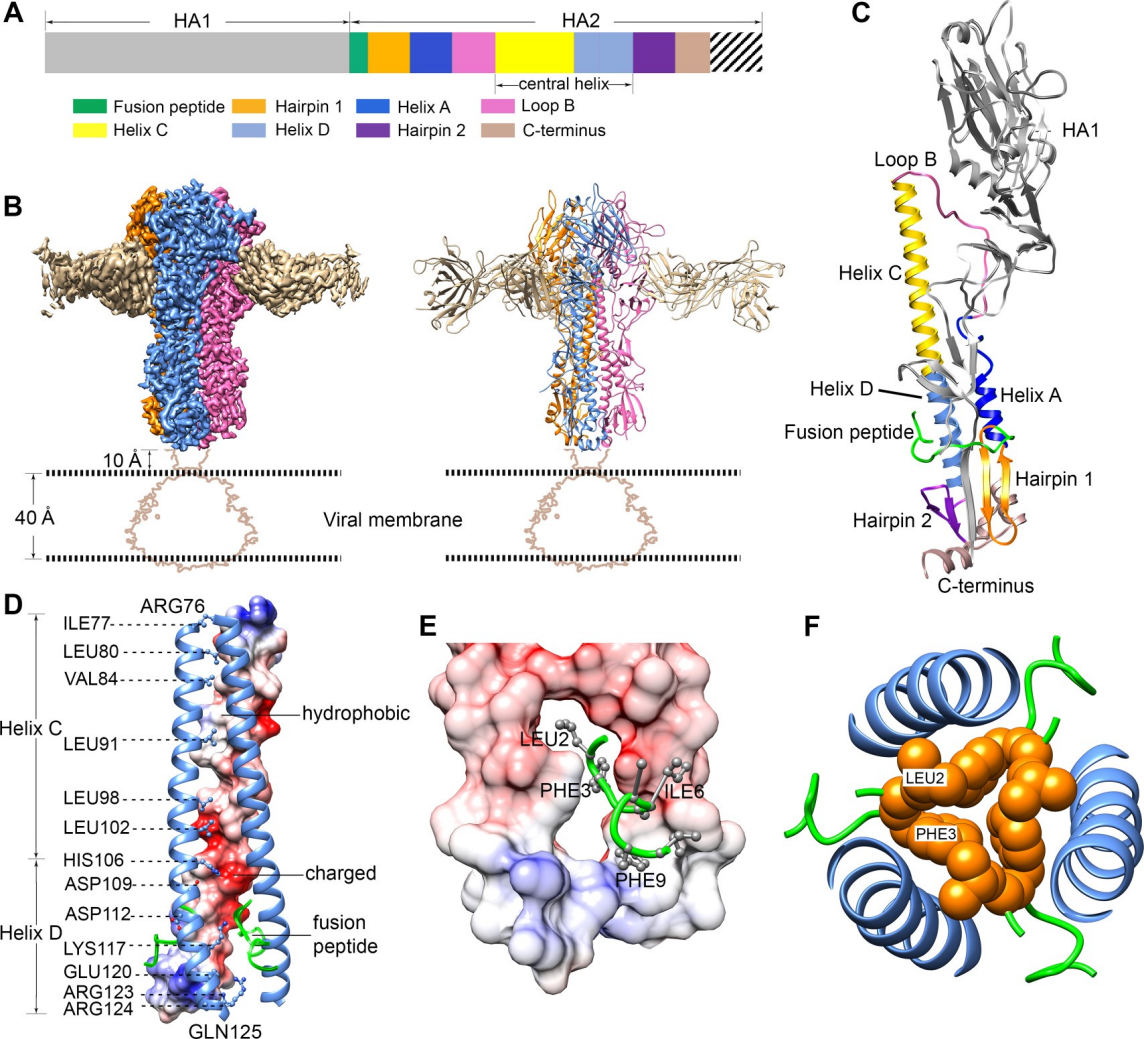
Cryo-EM structure of the influenza virus HA in complex with Fab F005-126 (HA-Fab) at pH 7.8. **(A)** A schematic diagram showing the domain organization of the influenza virus HA. HA1 subunit (9–324), gray. Fusion peptide (1–20), green. Hairpin 1 (21–38), orange. Helix A (39–56), blue. Loop B (57–75), hot pink. Helix C (76–105), yellow. Helix D (106–125), cornflower blue. Hairpin 2 (126–141), purple. C-terminus (142–172), brown. The transmembrane domain and the cytoplasmic tail (173–222) are represented as stripe lines. **(B)** Surface-shadowed (left) and ribbon diagrams (right) showing the 2.8 Å cryo-EM structure of the HA-Fab at pH 7.8. The map is contoured at 8 σ. The disordered transmembrane domain and the bound detergent micelle are visible only at a lower map contour level, and the profile of the corresponding part at a contour level of 3 σ is therefore indicated in the diagram by brown lines. The three HA1/HA2 heterodimers are colored hot pink, cornflower blue and orange, respectively. The bound Fabs are colored tan. **(C)** Ribbon diagrams showing the structure elements of a HA1/HA2 heterodimer. The structure elements are labeled and colored the same as in (**A**). **(D)** Ribbon and surface diagrams showing the central helices. Side chains of the residues in the core of one central helix are shown in balls and sticks. The surface of one central helix is shown and colored according to the surface electrostatic potentials, with blue representing positive electrostatic potential and red representing negative electrostatic potential. The ribbons of the fusion peptides are colored green. **(E)** Ribbon and surface diagrams showing one fusion peptide in the surface pocket between the Helix Ds. The surface is colored according to the surface electrostatic potentials. The backbone of the fusion peptide is colored green, and the sidechains of the hydrophobic residues are colored gray. **(F)** Ribbon diagrams showing the hydrophobic core constituted by the hydrophobic fusion peptide terminal residues. The backbone of the fusion peptide is shown in green. The N-terminal hydrophobic residues of the fusion peptide are shown as orange spheres. The central helices are colored cornflower blue.

The HA-Fab complex at pH 7.8 (HA-Fab-pH 7.8) was determined at a resolution of 2.8 Å (Figs 1 and S4). Atomic models of the HA ectodomain and the bound Fabs were built (Fig 1B and 1C). The transmembrane domain of the HA is flexible and was excluded from the final structure model (Figs 1B and S4). The cryo-EM structure of the HA at pH 7.8 is highly similar to the previously determined crystal structure of the HA ectodomain from an H3N2 virus (PDB accession number: 3WHE) [24]. Superimposition of the cryo-EM structure with the crystal structure shows an r.m.s.d. of 0.5 Å between the 1470 aligned C_α_ atom pairs of the HAs. Each HA trimer has three bound Fab F005-126s with each Fab F005-126 cross-linking two HA protomers. The bound Fab F005-126 has direct interactions with residues 171–173, 239, 240 of one HA protomer and residues 91–92, 270–273, 284–285 of a neighboring protomer (S5 Fig). The three central helices of the HA trimer intertwine to form a left-handed triple-stranded coiled coil (Fig 1D). Each central helix can be approximately divided into N-terminal (residues 76–105, Helix C) and C-terminal (residues 106–125, Helix D) segments (Fig 1D). The N-terminal Helix Cs are tightly packed through extensive hydrophobic interactions (Fig 1D). In addition, wrapping by the HA1 head should further stabilize the membrane-distal Helix Cs. Unlike the canonical coiled coil, the C-terminal Helix Ds are loosely packed and do not have a hydrophobic core. Instead, Helix D has many charged residues facing the three-fold axis of the HA (Fig 1D). The three Helix Ds are tethered together at the membrane-proximal ends, which bend and rotate anticlockwise by 3.5 degrees compared to a modeled coiled coil generated from the parameters of the Helix Cs (S6 Fig). The fusion peptide wraps around the N-terminal fragment of HA1 (residues 9–19 of HA1, HA1-N) and forms a hydrogen bond with residue His17 of HA1-N. Hydrophilic pockets are formed between the Helix Ds (Fig 1D and 1E). The hydrophobic distal ends of the fusion peptides insert deeply into the pockets between the Helix Ds and form a hydrophobic core of residues Leu2 and Phe3 (Fig 1E and 1F).

### Cryo-EM reveals three conformational states of HA-Fab complex at pH 5.2

**Fig 2:**
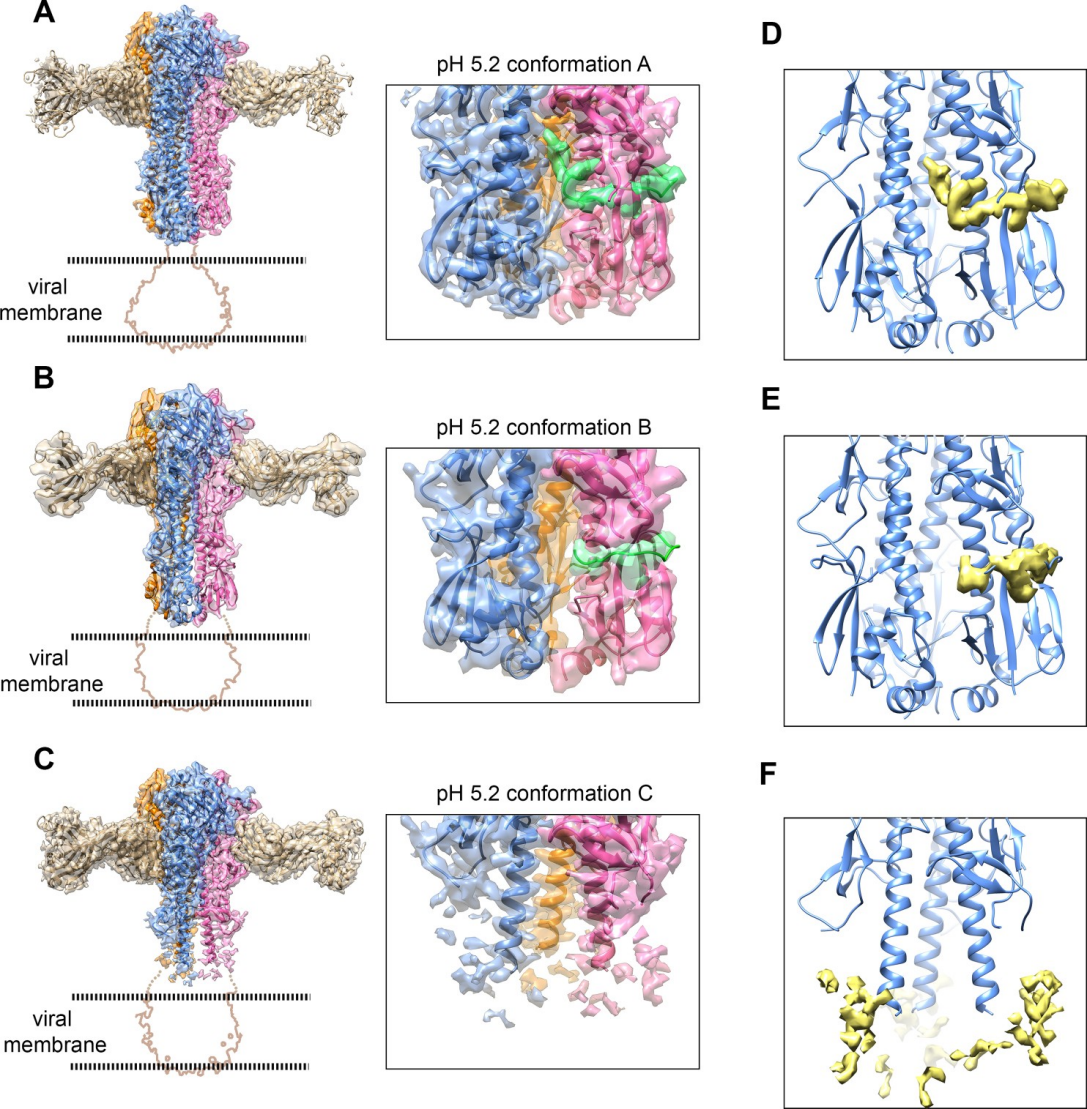
Structures of the HA-Fab complex at pH 5.2 showing the release of the fusion peptide. **(A** to **C)** Surface-shadowed and ribbon diagrams showing the HA-Fab-pH 5.2 conformation A **(A)**, conformation B **(B)** and conformation C **(C)**. Semitransparent surfaces displayed with the backbone ribbons in the density maps. The density maps are contoured at 7 σ **(A)**, 11 σ **(B)** and 7 σ **(C)**, respectively. The profiles of the disordered transmembrane domain and the bound detergent micelle (map contoured at 3 σ) are indicated by brown lines. The fusion peptides are colored green. The zoomed-in views show the densities around the fusion peptides. **(D** to **F)** Surface-shadowed and ribbon diagrams showing the residue densities of the “zero out” maps for the three low pH conformations. The “zero out” maps were calculated by setting the values of the map grid points within a radius of 2.5 Å of each fitted model atom to zero [52]. The fusion peptide (residues 1–20 of the HA2) was excluded for all the calculations. The “zero out” residue density maps are colored yellow and contoured at 7 σ **(D)**, 11 σ **(E)** and 7 σ **(F)**, respectively.

We obtained three major conformations of the HA-Fab complex at pH 5.2 (HA-Fab-pH 5.2 conformations A, B and C) through 3D classifications and refinements (Figs 2, S7 and S8). The structures of the three major conformations were determined at resolutions of 3.0 Å, 4.2 Å and 3.4 Å, respectively.

The HA-Fab-pH 5.2 conformation A has a highly similar structure to that of HA-Fab-pH 7.8 (S9 Fig) with an r.m.s.d of 0.3 Å between the 1470 aligned C_α_ atom pairs of the HAs. The small r.m.s.d values (pH 5.2 conformation A and pH 7.8: 0.25 Å; pH 5.2 conformation B and pH 7.8: 0.53 Å; pH 5.2 conformation C and pH 7.8: 0.36 Å) between the HA1 heads of different conformations indicated that the globular HA1 heads of the HA-Fab-pH 5.2 conformations B and C also remained unchanged, as expected (S9 Fig). The epitopes recognized by F005-126 at pH 7.8 and at pH 5.2 are the same and are highly similar to these observed in the crystal structure of HA and Fab F005-126 (S5 Fig). However, the HA-Fab-pH 5.2 conformations B and C showed significant differences in the stem region from those of the HA-Fab-pH 7.8 and the HA-Fab-pH 5.2 conformation A (Figs 2, S9 and S10).

### The fusion peptide is released in the conformation B of the HA-Fab complex at pH 5.2

The densities of the central helices in the HA-Fab-pH 5.2 conformation B are clearly visible, indicating that the central helix coiled coil is still stable and ordered in the structure (Figs 2B, S7 and S10C). The densities of the fusion peptides in the HA-Fab-pH 5.2 conformation B are largely absent in the hydrophilic pockets of the central helices (Fig 2B). These observations suggest that dropping the pH induced the release of the fusion peptides, which was also indicated by the aggregation of the particles (S2 and S3 Figs). In addition, the densities of β Hairpins 1 and 2, the C-terminus of the HA2 stem and HA1-N become smeared in the HA-Fab-pH 5.2 conformation B (S10 Fig), suggesting disorder in these regions. Although structural details of the residues in the disordered regions are missing, envelopes for the domains and secondary structures of the disordered region can still be defined. Rigid-body refinements of the structure segments in the stem region indicate no significant domain movements. The release of the fusion peptide can be further confirmed by a “zero out” map calculated between the map and the atomic model without the N-terminus of the fusion peptides (residues 1–20), which shows no obvious residue density in the fusion peptide binding pockets (Fig 2E).

### The central helices are unwound in the conformation C of the HA-Fab complex at pH 5.2

**Fig 3:**
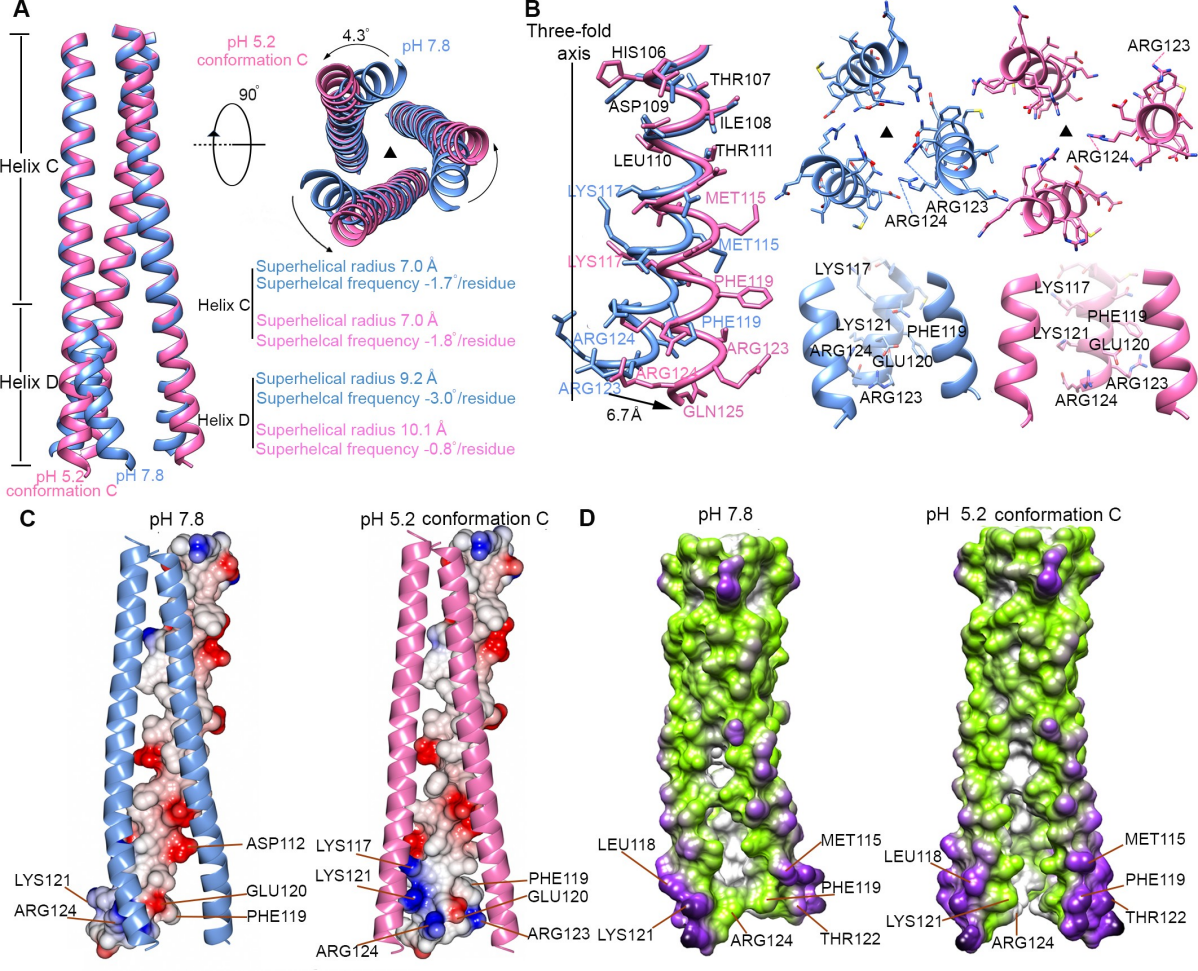
Structure comparisons of the pH 7.8 and pH 5.2 conformations. **(A)** Structure superimpositions of the central helices in neutral (cornflower blue) and low pH conformations (hot pink). Side view (left) and bottom-up view (right) are shown. The coiled-coil parameters of the Helix Cs and the Helix Ds in neutral and low pH conformations are listed under the top view. The rotation directions of the membrane-proximal ends are indicated by black arrows in the bottom-up view. The rotation angles of the helices are measured with the membrane-distal ends as the pivot point. **(B)** Structure comparisons between the Helix Ds in neutral (cornflower blue) and low pH (hot pink) conformations showing the changes in the residue side chains. **(C)** Ribbon and surface-rendered diagrams showing the surface electrostatic potential changes of the central helices upon pH change. The surface is colored according to the surface electrostatic potential with positive charges colored blue and negative charges colored red. **(D)** Surface-rendered diagrams showing the changes in surface and inner cavity of the central helices upon pH change. The surface is colored from white to green to purple according to the distances from the voxels to the three-fold axis.

The densities around the stem region of the HA-Fab-pH 5.2 conformation C are highly disordered, and atomic models were built only for the head and the central helix (Figs 2C, S7 and S10D). The central helices of the HA-Fab-pH 5.2 conformation C adopt a straight and extended conformation rather than the bent form observed in other conformations (Fig 3A). Further analysis showed that conformational changes occur mainly in the Helix Ds, as indicated by the calculated r.m.s.d values (0.4 Å between the 90 aligned C_α_ atom pairs of the Helix Cs *vs* 4.0 Å between the 60 aligned C_α_ atom pairs of the Helix Ds, Fig 3A). The membrane-proximal ends of the central helices have a shift of 6.7 Å compared to those in the pH 7.8 and other pH 5.2 conformations, which corresponds to an anticlockwise rotation of 4.3 degrees around the three-fold axis when viewed from the bottom of the central helices and with the membrane-distal end of the three-fold axis as the pivot point (Fig 3A and 3B). The coiled-coil parameters of the Helix Cs and Helix Ds in different conformations were calculated and compared. The coiled-coil parameters of the superhelical radius and superhelical frequency calculated for the Helix Cs of the pH 7.8 conformation are 7.0 Å and -1.7° per residue, respectively, while these for the Helix Cs of the HA-Fab-pH 5.2 conformation C are 7.0 Å and -1.8° per residue, respectively. However, the coiled-coil parameters of the superhelical radius and superhelical frequency calculated for the Helix Ds of the pH 7.8 conformation are 9.2 Å and -3.0° per residue, respectively, whereas those for the Helix Ds of the HA-Fab-pH 5.2 conformation C are 10.1 Å and -0.8° per residue, respectively (Fig 3A). Both the increase in the superhelical radius and the decrease in the superhelical frequency of the Helix Ds indicate the unwinding of the coiled coil. Splaying of the helices were also observed in two trimer tag stabilized HA2 structures [27,28], in which the Helix Ds has a splaying angle of approximately 30 degrees [27] (S11 Fig) and may present an intermediate to the postfusion state or an irrelevant state caused by the trimer tag.

**Fig 4:**
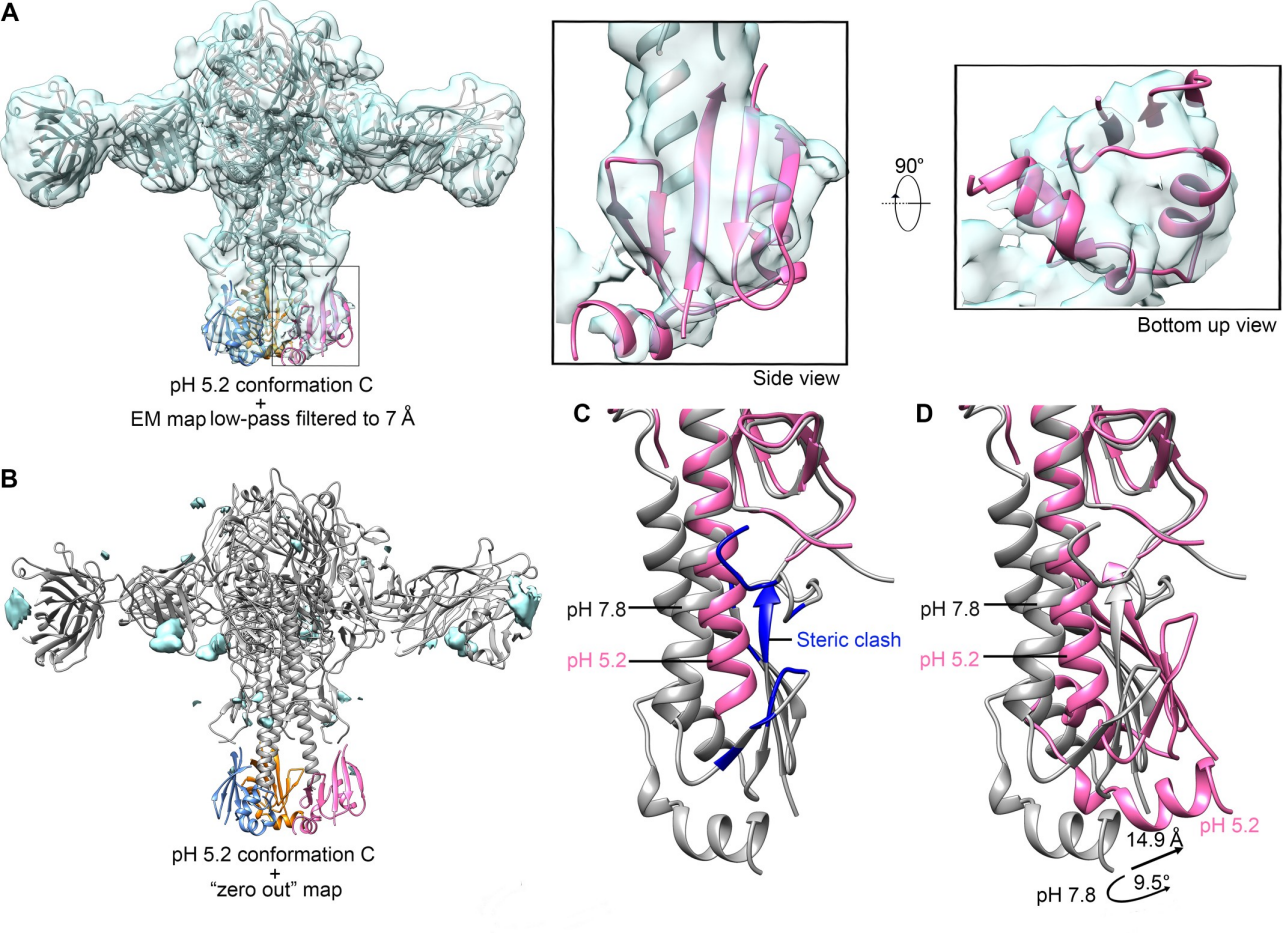
Structure changes in the stem region of the pH 5.2 conformation C. **(A)** Ribbon and surface rendered diagrams showing the fitted beta sheet, C-terminal helices of the stem region (residues 23–37 and 126–172 of HA2 subunits and 9–17 of HA1 subunits) in the pH 5.2 conformation C density map low-pass filtered to 7 Å. The fitted structures in the HA density are colored hot pink, cornflower blue and orange, respectively. The head and central helices are shown in gray. The low-pass filtered pH 5.2 conformation C density map is contoured at 6 σ and shown as a semi-transparent surface. Zoom-in views of the fitted region are shown at the right. The fitting was done in Chimera by treating the beta sheet and the C-terminal helices of the stem region as a rigid body and by maximizing the density values around the fitted atoms [52]. **(B)** Models are shown as the same as in (A) with a “zero-out” density map of the pH 5.2 conformation C shown as solid surfaces in cyan. The “zero-out” density map was calculated by setting the density value within 3 Å around the atoms of the model to zero. **(C)** Models of pH 5.2 conformation C (hot pink) and pH 5.2 conformation A (gray) are superimposed by using the head domains. The steric clashes between the central helices of the pH 5.2 conformation C and the stem region (fusion peptide and beta sheets) of the pH 5.2 conformation A are shown in blue. **(D)** Structure comparisons of the stem regions under different pH conditions. The stem region rotates and shifts upon the decrease of the pH. The shift of the C terminus is approximately 15 Å and the shift of the center of mass is approximately 7 Å. The rotation of the C terminus with the membrane-distal end of each central helix as the pivot point (residue Arg76) is approximately 9.5 degrees.

**Fig 5:**
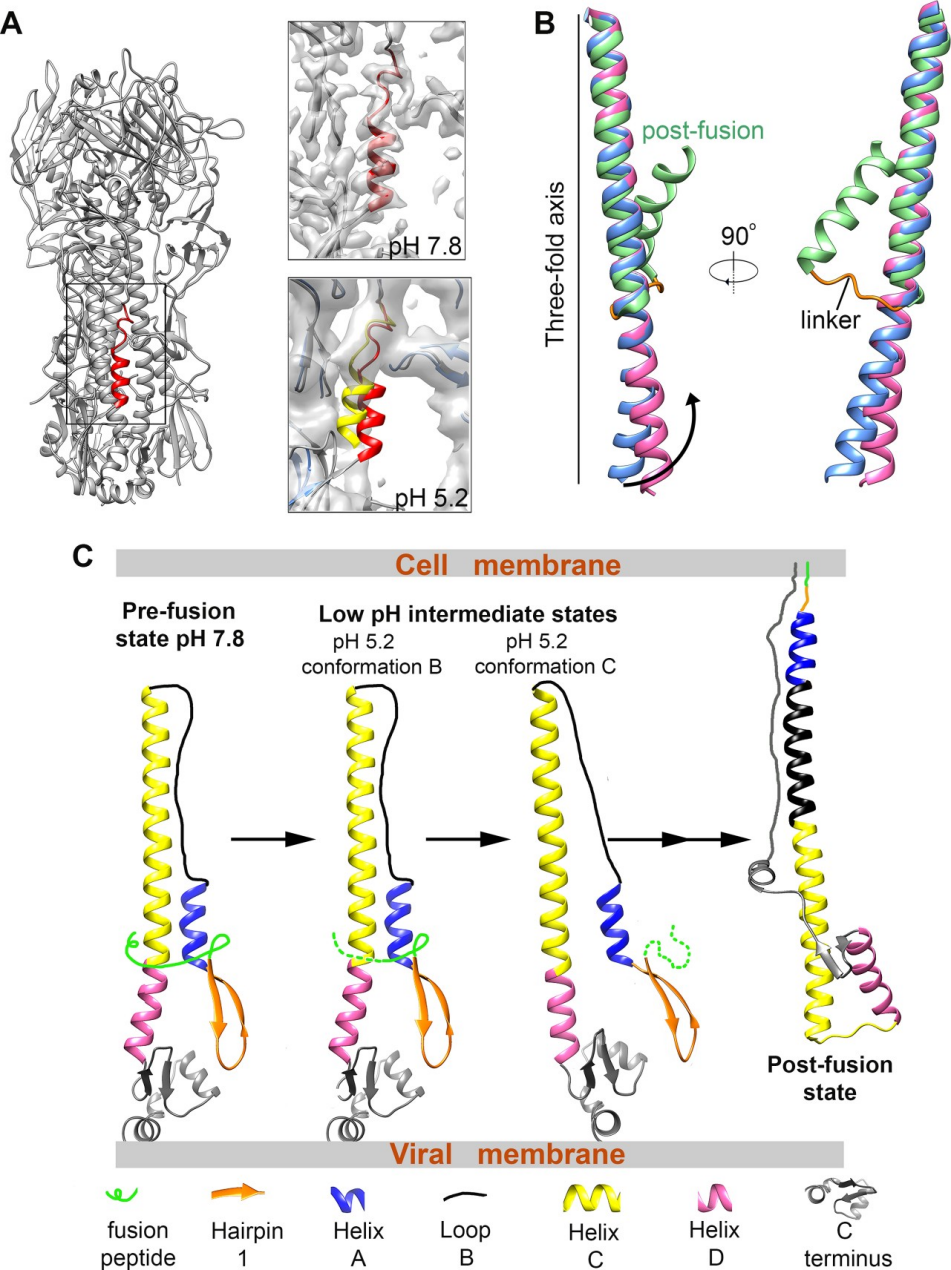
Molecular basis of the pH-induced conformational changes of HA. **(A)** Structure comparisons of the Helix As in neutral and low pH conformations. Left: Ribbon diagrams showing the position of Helix A in the stem region. Helix A is colored red. Right: Zoomed-in views showing the Helix As in neutral (red) and low pH (yellow) conditions. The ribbons of the stem are colored dark gray for the neutral pH conformation and blue for the low pH conformation. **(B)** Structure comparisons of the neutral pH (cornflower blue), low pH intermediate (hot pink) and postfusion (green, PDB accession: 1HTM) HA central helices. The linker conducting helix to loop conformational change in the post-fusion transition is colored orange. **(C)** A schematic diagram illustrating the low pH-induced structural transition of HA.

The conformational changes enlarged the distances between the membrane-proximal ends of the central helices and created an inner cavity with a volume of approximately 1400 Å^3^ (S12 Fig). The interactions between the helices observed in the neutral conformation are disrupted (Fig 3B). In addition, the conformational changes of the central helices completely alter the properties of the fusion peptide binding pockets and the inner and outer surface of the central helices (Figs 3C, 3D and S13). Furthermore, the extended Helix C would have steric clashes with the fusion peptides in the conformation at pH 7.8 (Fig 4), indicating that the fusion peptides could not return to their neutral pH position and conformation. Similarly, the extended Helix D would have steric clashes with the outer Helix A, Hairpin 2 and HA1-N (Fig 4), suggesting that Helix A and the β sheet constituted by Hairpins 1 and 2 and HA1-N must undergo conformational changes to adapt to the extended Helix D. The densities around the Helix A indicate that its C-terminal end should have a clockwise shift (Figs 5A and S14). The major epitopes recognized by several characterized broadly neutralizing antibodies of HA are around Helix A of HA2 in the stem region [20,25,29–32]. Antibody binding to the nearby epitopes could either prevent or hinder the conformational changes of Helix A, which is required for the conformation change in the central helices. Accordingly, the conformation change in the central helices will affect the binding of stem specific antibodies as well, especially for these targeting around the Helix A. As indicated by our ELISA assays with the antibody 31.a.83 that mainly targets Helix A [32], the binding of 31.a.83 to HA was significantly reduced over the time course of low pH treatment (S15 Fig). The densities for the β sheet and the C-terminal helices are highly disordered in the cryo-EM map. However, the envelope of these regions could be clearly defined when the map was low-pass filtered to a resolution of 7 Å (Fig 4). The fitting of β Hairpins 1, 2 and the C-terminus of the HA2 stem and the HA1-N into the cryo-EM map as rigid bodies showed a rotation of 9.5 degrees around the three-fold axis and a shift of approximately 15 Å compared to those in other conformations (Fig 4).

## Discussion

Our observations of the low pH induced intermediate states suggest that the pre-postfusion transition involves at least two intermediate steps, including one step in which the fusion peptides are released from the surface pockets and a subsequent step in which the central helices unwind (Fig 5B and 5C). The surface grooves and the larger inner cavity in the stem region of the late intermediate conformation could provide new sites for designing universal vaccines and inhibitors to influenza viruses. FRET studies also suggested the existence of a stable intermediate state of HA in the low pH induced transition, in which the fusion peptide is far away from the central helices [14].

Recently, low pH induced intermediates of HA ectodomain have been reported, including one form with a dilated HA head, a second form with a dilated HA head, unwound central helices and a third form with an extended HA2 [33]. In our research, we used a neutralizing antibody, F005-126, to bind the HA head and found that the HA head remains tightly packed upon low pH treatment, indicating that this antibody prevents the dilation of HA head even after an incubation of 30 minutes at room temperature (see Materials and methods). The second form in that paper is quite consistent with our conformation C, as they both feature with unwound central helices. Of note, the conformation B in our research has not been reported. The conformation B has the released fusion peptides while the other domains remain in similar conformation with the neutral pH structure. Moreover, our research also suggests that release of the fusion peptide and unwinding of the central helices are likely to be independent to the dilation of the HA head which is a prerequisite for subsequent conformational change including the extending of HA2. In addition, unwinding of the central helices is not constrained by C-terminal transmembrane domain of HA since we used a full-length HA in our research. It’s likely that our strategy of using a HA head specific antibody helps to stabilize this intermediate state which is then captured by cryo-EM. However, we cannot rule out the possible effects of Fab binding on the sequence of the events happening in the pre-post fusion transition of HA and possible irrelevant conformation changes introduced by Fab binding as have been observed in other studies [25,32,34–36]. Further studies under physiological conditions are yet to be performed in the future towards a more comprehensive understanding of influenza virus entry.

Our study showed that after being stabilized by Fab F005-126, the interface between the HA1 globular head and Fab F005-126 remained intact even after low-pH treatment for 30 minutes (S5 Fig). This is consistent with the ELISA results (S15C and S15D Fig). The antibody stabilizes the HA head and prevents further conformation change to the post-fusion state. HA head could be a promising target for neutralizing antibodies not only by blocking interaction with receptor but also by preventing the low pH induced structure transition.

In summary, we reported two intermediate states of influenza HA at the low pH-induced transition, providing structural snapshots of HA in the highly dynamic process during virus-host cell membrane fusion. Our work, together with others, could potentially provide a framework for antiviral drug and antibody development.

## Materials and methods

### Gene synthesis and cloning

The genes encoding the HA of the A/Hong Kong/1/1968 strain (H3N2) (UniProt ID: Q91MA7), the neutralizing antibody F005-126 (GenBank accession number: AB848924 for the V_H_ region of the H chain, the CH_1_ region of the H chain is from the expression vector and GenBank accession number: AB848925 for the L chain) and the stem specific antibody 31.a.83 (PDB accession number: 5KAQ) were synthesized (Qinglan Biotech co., Wuxi, China) with codon optimization for expression in mammalian cells. The synthesized HA gene was cloned into the pCMV vector, which introduced a strep tag at the C-terminus of the recombinant HA. The HA ectodomain part (residues 17–519) was also inserted into the pCMV vector with the N-terminal gp67 signal peptide and the C-terminal strep tag. The antibody was cloned into a specialized vector with a preinserted region encoding a signal peptide (GWSCIILFLVATATGVHS) and a human IgG1 antibody constant region (Fc) [37].

### HA expression and purification

F005-126 binds the globular head of HA, crosslinks two HA1 protomers, and was suggested to function through preventing the low pH-induced conformational changes of HA [24]. We produced the antibody F005-126 and the recombinant full-length HA in HEK293F cells. The antigen-binding fragment (Fab) of F005-126 was prepared by protease cleavage of F005-126, and the HA-fab complex was assembled *in vitro* from purified Fab and HA.

The plasmids were extracted and purified using plasmid extraction kits (Tiangen, Inc., Beijing, China, DP117). HEK293F cells were grown in SMM 293-TII medium (Sino Biological Inc., M293TII-1) supplemented with 0.5% FBS. For one liter of cell culture, 2 mg of purified plasmid was preincubated with 8 mg of polyethylenimine (PEI) (Polysciences, 24765–2) for 20 minutes, and then the cells at a density of 2.5–3×10^6^ cells/ml were transiently transfected with the plasmid-PEI mixture. The transfected cells were harvested 48 hours posttransfection by centrifugation at 1000×g for 5 minutes. The supernatant was discarded, and the cell pellet was resuspended in lysis buffer (20 mM HEPES pH 7.8, 150 mM NaCl) and sonicated. The cell membrane was collected by ultracentrifugation at 100,000×g for 1 hour at 4°C. The membrane pellet was resuspended with 1% (w/v) lauryl maltose neopentyl glycol (LMNG) (Anatrace, NG310) in lysis buffer and incubated at 4°C for 2 hours. The extraction was then ultracentrifuged at 150,000×g for 30 minutes. The supernatant was collected and applied to Strep-Tactin Superflow beads (IBA, 2-1206-010), which were precooled at 4°C and balanced with a buffer containing 20 mM HEPES at pH 7.8, 150 mM NaCl and 0.003% LMNG. The beads were washed with balancing buffer. The recombinant HA was eluted with the balancing buffer containing 5 mM d-desthiobiotin (Sigma, D1411). The eluted sample was concentrated and treated with trypsin (Sigma, T1426) for 12 hours in a buffer containing 50 mM Tris-HCl at pH 8.0, 20 mM CaCl_2_ and 0.003% LMNG. Trypsin was added at a trypsin:HA ratio of 1:500 (w/w). Then, protease inhibitor cocktail (Roche, 04693116001) was added, and the cleaved HA was purified through size exclusion chromatography (SEC) with a Superose-6 Increase column (GE Healthcare, 29-0915-96) running in a buffer containing 20 mM HEPES at pH 7.8, 150 mM NaCl and 0.003% LMNG.

### HA ectodomain expression and purification

The plasmid preparation and transfection procure were the same as for the full-length HA. The supernatant containing the HA ectodomain was collected by centrifugation 48 hours posttransfection. After concentration and buffer exchange, the supernatant was loaded onto Strep-Tactin Superflow beads. The buffer used was 20 mM HEPES at pH 7.8 with 150 mM NaCl. The recombinant HA ectodomain was eluted with the same buffer supplemented with 5 mM d-desthiobiotin.

### F005-126 and 31.a.83 expression and purification

Preparation of the antibody expression plasmids followed the same procedure as for HA expression. One liter of HEK293F cell culture was transfected with 1.5 mg of plasmid encoding the F005-126 heavy chain, 1.5 mg of plasmid encoding the F005-126 light chain and 12 mg of PEI. The supernatant containing F005-126 was collected by centrifugation 72 hours posttransfection. After concentration and buffer exchange, the supernatant was loaded onto a protein A Sepharose column (GE Healthcare, 17-0780-01). The column was washed with a buffer containing 20 mM HEPES at pH 7.8 and 150 mM NaCl. The bound antibody was eluted with 0.1 M glycine at pH 3.5. The eluted sample was immediately neutralized by 1 M Tris at pH 9.0. To prepare the Fab, GST-tagged HRV3C was added to the neutralized elution and incubated at 4°C overnight. The protease was removed by affinity purification using glutathione Sepharose 4B beads (GE Healthcare, 17-0756-01). The cleaved free Fc and the uncleaved F005-126 were removed using a protein A Sepharose column. The expression and purification of 31.a.83 was similar to that of F005-126. The stem specific antibody 31.a.83 in full length was used in the ELISA assay instead of its Fab part.

### HA-Fab F005-126 complex purification

The purified HA and Fab were mixed at a molar ratio of 1:3 (HA: Fab). After incubation at 4°C overnight, the complex was purified by size exclusion chromatography with a Superose-6 Increase column (GE Healthcare, 29-0915-96) running in a buffer containing 20 mM HEPES at pH 7.8, 150 mM NaCl and 0.003% LMNG. Peak fractions containing both HA and Fab F005-126 were collected and concentrated to ~ 0.4 mg/ml for cryo-EM sample preparation.

### Low pH treatment of the HA and Fab F005-126 complex

The pH of the purified HA-Fab F005-126 complex was adjusted to pH 5.2 by adding 0.5 M sodium acetate at pH 4.6. The final pH was verified with a pH meter. The sample was incubated at room temperature for 30 minutes and was then loaded directly onto grids for cryo-EM sample preparation.

### Trypsin susceptibility assay of the low pH treated ecto HA-Fab F005-126 complex

The pH of the purified ecto HA-Fab complex sample was adjusted to 5.2 by the addition of 0.5 M sodium acetate at pH 4.6. Aliquots were taken as samples after incubation at room temperature for 30 minutes and were immediately neutralized by 1 M Tris (pH 9.0). Trypsin was then added to the samples at a ratio of 1:20 (trypsin:HA, w/w). After trypsin digestion at 37°C for 1.5 hours, the samples were mixed with the reducing loading buffer, boiled at 96°C for 5 minutes and then analyzed by using an SDS-PAGE gel. The amount of HA loaded in each lane was adjusted to be the same.

### Detection of the low pH induced conformational changes in the stem with a stem specific antibody

HA protein was purified and trypsin cleaved by following the same procedure as mentioned above. The purified and trypsin cleaved HA was coated directly onto a 96-well ELISA plate overnight at 4°C. For each microplate well, 100 ng of HA was added. The coated HA was treated by a buffer at pH 5.2 before or after the addition of the stem specific antibody. The stem specific antibody 31.a.83 at a concentration of 10 μg/ml was serially diluted and added to the microplate well. Low-pH treated HA samples were neutralized to pH 7.8 before the incubation with the stem specific antibody 31.a.83. Detection of 31.a.83 was done by using an anti-human immunoglobulin G conjugated with the horseradish peroxidase (Promega, W4038). Low-pH treatment and antibody incubation were performed under 37°C. TMB (3,3′,5,5′-tetramethylbenzidine) substrate (CWBIO, CW0050S) was used for the horseradish peroxidase-based detection and the reaction was stopped by adding H_2_SO_4_ with the concentration of 0.5 M. Signal of the horseradish peroxidase product was detected by measuring their absorbance at 450 nm. HA-Fab complex was purified with a same procedure as mentioned above and the ELISA procedure was similar as those for the HA samples.

### Dynamic light scattering analysis of the HA-Fab F005-126 samples at pH 7.8 and at pH 5.2

Dynamic light scattering (DLS) analysis of the samples at pH 7.8 and at pH 5.2 were performed by using the DynaPro NanoStar (Wyatt) within a time course of 30 minutes under 25°C. Purified HA-Fab complex was filtered with 0.22 μm centrifugal filters before being loaded into disposable cuvettes and the sample was mixed gently by using a pipette 2 minutes before each measurement, which took a period of 50 seconds for 10 acquisitions. Scattering data were analyzed with the software DYNAMICS 7.1.7.

### Cryo-EM data collection

Three-microliter aliquots of purified complex sample, at a concentration of 0.4 mg/ml, were applied to glow-discharged holey carbon grids (Quantifoil, Cu 400 mesh, R1.2/1.3). The grids were blotted and then plunged into liquid ethane by using a Vitrobot Mark IV (Thermo Fisher).

Images of the HA-Fab complex at neutral pH and at low pH were recorded using a Titan Krios electron microscope (Thermo Fisher) operating at an acceleration voltage of 300 keV and equipped with a GIF Quantum energy filter (slit width 20 eV) and a Gatan K2 Summit camera. Images were recorded at a defocus range of -1 μm to -3.5 μm with a pixel size of 0.665 Å. Each image was dose-fractionated into 32 movie frames at a dose rate of 8.2 counts per physical pixel per second, with a total exposure time of 8 s and a frame exposure time of 0.25 s, resulting in a total dose of ~50 electrons per Å^2^. AutoEMation2 was used for the data collection [38].

### Image processing

The image processing procedures are summarized in S4 and S8 Figs. Data collection and image processing statistics are listed in S1 Table. All 32 frames in each stack were aligned and summed by using the program MotionCor2 and were binned by a factor of 2 pixel × 2 pixel [39]. The CTF parameters were determined with the program Gctf [40]. Particles were automatically selected using Gautomatch [41]. Subsequent 2D and 3D analyses were performed using RELION [42].

For the dataset of the HA-Fab complex at pH 7.8, 567,365 particles were selected after several rounds of 2D classifications. The crystal structure of the HA ectodomain and Fab F005-126 complex (PDB accession number: 3WHE) was low-pass filtered to 40 Å and used as the initial model. After one round of 3D classification, 359,922 particles were selected and subjected to 3D refinement. To improve the map quality of the ectodomain, a mask covering the ectodomain was applied for the last round of the refinement, and the 3D refinement cycles were performed until convergence between the iterations, yielding a 2.8 Å density map. The 3D classifications and refinements were conducted with the C3 symmetry imposed.

For the dataset of the HA-Fab complex at pH 5.2, 579,459 particles were selected after several rounds of 2D classifications. The density map of the HA-Fab complex at pH 7.8 was used as the initial model. After one round of 3D classification, 486,746 particles were selected and subjected to 3D refinement. Then, two parallel rounds of 3D classification were conducted, yielding six classes and five classes, respectively. Different conformations were observed. Particles from classes with similar features to those of the HA-Fab conformation at pH 7.8 were combined and subjected to final 3D refinements and reconstructions, yielding a 3.0 Å density map (pH 5.2 conformation A). Particles from classes showing obviously different conformations in the stem region were combined and subjected to another round of 3D classification, yielding six classes. One of these classes showed similar overall features to those of the pH 7.8 conformation except for the fusion peptides. Particles from this class were selected and subjected to final 3D refinements and reconstructions, yielding a 4.2 Å density map (pH 5.2 conformation B). Particles from two classes showing conformational changes in the central helixes were combined and subjected to final 3D refinements and reconstructions, yielding a 3.4 Å density map (pH 5.2 conformation C). Other classes contained only a few particles and were discarded.

Prior to visualization, all density maps were sharpened by applying a negative B-factor and corrected for the modulation transfer function (MTF) of the detector by using RELION. The reported resolutions are based on the gold-standard Fourier shell correlation (FSC) 0.143 criterion [43]. Directional FSC plots for the reconstructions were calculated on the 3DFSC server [44]. Variations in local resolution were estimated by using RELION.

### Model building and structure refinement

Model building and structure refinement statistics are listed in S1 and S2 Tables.

The crystal structure of HA-Fab F005-126 (PDB ID: 3WHE) was fitted into our cryo-EM density maps in Chimera and was used as a starting model. The structure models were built and adjusted in COOT [45] and were refined by using PHENIX [46] real-space refinement with secondary structure and geometry restraints. MolProbity [47] and EMRinger [48] were used to evaluate the final refined models.

The atomic model of the stem region of the pH 5.2 conformation C was not built due to the weak density. However, a bulky density was observed when the density map was low-pass filtered to 7 Å. Thus, we fitted the stem region of the pH 7.8 structure into the density map of the pH 5.2 conformation C as a rigid body (Fig 4). Specifically, the density map of the pH 5.2 conformation C was applied with a B-factor of -150 Å^2^ and low-pass filtered to 7 Å. The refined model of the pH 5.2 conformation C was converted to a 7 Å density map, and the corresponding density was extended by 2 Å and then subtracted from the low-pass filtered density map of the pH 5.2 conformation C. The model containing residues 23–37 and 126–172 of the HA2 and residues 9–17 of the HA1 was fitted into the subtracted density map as a rigid body in Chimera.

The coiled-coil parameters were calculated using the program Coiled-coil Crick Parameterization (CCCP) [49]. A modeled three-strand coiled-coil model (S6 Fig) was generated for comparisons by using CCCP with the coiled-coil parameters of the Helix Cs.

The steric clashes between the models were calculated with Chimera (Fig 4). Two atoms are considered to have clashes if the overlap score between them is more than 0. The overlap score is defined as the sum of two van der Waals (VDW) radii minus the distance between them and minus an allowance (0.4 Å) for potentially hydrogen-bonded pairs[50].

All figures were generated with Chimera [50].

## Supporting information

S1 Fig Antibody F005-126 prevents the low-pH induced conformation change of HA. The ectodomain of HA is digested by trypsin after the incubation of 30 minutes under pH 5.2 with or without Fab F005-126. The bound Fab F005-126 prevents the pre-postfusion transition of the HA and renders the HA trypsin resistant.
(TIF)

S2 Fig Representative cryo-EM micrographs and 2D class averages of the HA-Fab complex under different pH conditions. **(A)** A representative raw micrograph (top) and 2D class averages (bottom) of the HA-Fab complex at pH 7.8. **(B)** A representative raw micrograph (top) and 2D class averages (bottom) of the HA-Fab complex at pH 5.2. Scale bars in the raw micrographs represent 50 nm. Scale bars in the 2D class averages represent 10 nm.
(TIF)

S3 Fig Dynamic light scattering analysis of the HA-Fab F005-126 complex at pH 7.8 and at pH 5.2. Curves measured at three time points were shown. The neutral pH sample is in a buffer containing 150 mM NaCl, 20 mM HEPES at pH 7.8 and 0.003% LMNG and the complex is at a concentration of 0.07 mg/ml. The low pH sample is in a buffer containing 150 mM NaCl, 20 mM HEPES at pH 5.2 and 0.003% LMNG and the complex is at a concentration of 0.07 mg/ml. (**A**) Size distribution of the HA-Fab complex at pH 7.8. (**B**) Size distribution of the low-pH treated HA-Fab complex.
(TIF)

S4 Fig Cryo-EM data processing of the HA-Fab complex at pH 7.8. **(A)** Cryo-EM data processing flowchart. See Materials and methods for details. **(B)** Local resolution map. **(C)** Particle orientation distribution. **(D)** Directional FSC plot for the reconstruction calculated on the 3DFSC server. Sphericity indicates the degree of anisotropy present in the reconstruction. Histogram indicates the portion of voxels with a particular resolution.
(TIF)

S5 Fig Space filling models showing the contacting interface of HA with Fab F005-126. The two HA protomers in the trimer are colored dark grey and light pink color, respectively. (**A**) The residues involved in direct interactions with Fab F005-126 in the reported crystal structure (PDB accession number: 3WHE) were shown in red color. (**B-C**) The contacting interface (in red) of HA with Fab F005-126 in different conformations. The contacting interface was calculated with Chimera. Two atoms are considered to have close contacts if the distance between them minus the sum of their van der Waals radii is less than 1.
(TIF)

S6 Fig Differences in the coiled coils of the central helices. The central helices at pH 7.8 (cornflower blue) superimposed with a model (coral) generated by using the coiled coil parameters of the Helix Cs. Side view (left) and bottom view (right) are shown. The Helix Ds are more open and have a larger twist compared to the simulated coiled coil. The significant structure differences between the simulated coiled coil and the central helices also indicate differences in the coiled coil parameters of the Helix Cs and the Helix Ds.
(TIF)

S7 Fig Cryo-EM data processing of the HA-Fab complex at pH 5.2. **(A-D)** Representative 2D class averages (**A**), local resolution (**B**), particle orientation distribution (**C**) and directional FSC plots of the HA-Fab reconstructions at pH 5.2 (**D**). Left, pH 5.2 conformation A. Middle, pH 5.2 conformation B. Right, pH 5.2 conformation C. The directional FSC plots for the reconstructions are calculated on the 3DFSC server. Sphericities indicate the degree of anisotropy present in the reconstructions. Histograms indicate the portion of voxels with a particular resolution.
(TIF)

S8 Fig Cryo-EM data processing flowchart of the HA-Fab complex at pH 5.2. See Materials and methods for details.
(TIF)

S9 Fig Structure comparisons between the conformations at different pHs. Structure superimpositions of the pH 5.2 conformation A (left, green), pH 5.2 conformation B (middle, gold) and pH 5.2 conformation C (right, hot pink) with the conformation at pH 7.8 (gray), respectively. The r.m.s.d. values between the 813 aligned C_α_ atom pairs of HA heads (residues 43–313 of HA1) of pH 5.2 conformation A, pH 5.2 conformation B and pH 5.2 conformation C with the conformation at pH 7.8 are 0.25 Å, 0.53 Å and 0.36 Å, respectively.
(TIF)

S10 Fig Cryo-EM densities of the selected representative regions. **(A-D)** Densities around the central helix (residues 76–125 of HA2) and the beta sheet (residues 9–18 of HA1, 21–38 and 126–141 of HA2) in the stem region of different conformations. Density maps are shown as meshes. Residue side chains are shown in balls and sticks with oxygen atoms colored red, nitrogen atoms colored blue and sulfur atoms colored yellow. The contour levels of the maps are listed under each conformation.
(TIF)

S11 Fig Structure comparisons of the HA central helices. The central helices of the pH 7.8 conformation (cornflower blue), the pH 5.2 conformation C (hot pink) and the headless HA (PDB accession number: 5CJQ), which could represent different stages in the pre-post transition, are compared. Side view (left) and bottom-up view (right) are shown, respectively. Position of three-fold axis in the bottom-up view is indicated by a black triangle. Possible subsequent conformational changes of the Helix Ds in the pre-post transition are indicated by the black arrows.
(TIF)

S12 Fig Changes in the inner cavity of the central helices upon low pH exposure. **(A)** Changes in the pore radius along the Z axis. **(B)** Comparisons between the inner cavities of the neutral and low pH conformations. Cavities colored green are only enough to adapt single water molecule. Cavities colored blue are large enough to adapt more than two water molecules [53].
(TIF)

S13 Fig Residue conservation analysis of the central helices. **(A)** Sequence alignments of the HA2s. The completely conserved residues are shown in white on a red background. The conserved residues are boxed. **(B)** Surface rendered diagrams showing the pH induced conformational changes of the conserved residues on the surface of the central helices. Residues completely conserved in both the group 1 and group 2 HA2s are colored black. Residues conserved in both group 1 and group 2 HA2s are colored dark gray. Residues not conserved are colored white. **(C)** Surface rendered diagrams showing the pH induced conformational changes of the conserved residues on the surface of the central helices of the group 2 HA2s. Residues completely conserved in the group 2 HA2s are colored black. Residues conserved in the group 2 HA2s are colored dark gray. Residues not conserved are colored white.
(TIF)

S14 Fig Surface rendered diagrams showing the changes in the stem region upon pH change. Voxels are colored according to their distances to the three-fold axis. All the maps were low-pass filtered to 8.3 Å. Positions of the Helix As and the fusion peptides are indicated.
(TIF)

S15 Fig ELISA assays to detect the low pH induced conformation changes in the stem region. The stem specific antibody 31.a.83 was serially diluted and detected for its binding to HA. Low pH treatments were taken under 37°C. (**A**) The epitope of 31.a.83 is shown in a space-filling model. The two HA protomers are colored dark grey and light pink, respectively. Residues involved in the epitope of 31.a.83 based on the reported structure (PDB accession number: 5KAQ) are colored red or blue. Residues in blue belong to Helix A. **(B)** 31.a.83 binding to the low pH treated HA. (**C**) Serially diluted 31.a.83 was added to the HA sample before low pH treatment and comparisons of 31.a.83 binding to the HA under neutral and low pH conditions by using the premixed samples. (**D**) 31.a.83 binding to the low pH treated HA-Fab F005-126 complex. All the measurements were repeated three times.
(TIF)

S1 Table Cryo-EM data collection, image processing and structure refinement statistics. (DOCX)

S2 Table Model building statistics. (DOCX)
