# Supplementary figures and images for: Structural intermediates in the low pH-induced transition of influenza hemagglutinin
Source: PLoS Pathog. 2020 Nov 30;16(11):e1009062. doi: 10.1371/journal.ppat.1009062 (PMC7728236; doi:10.1371/journal.ppat.1009062)

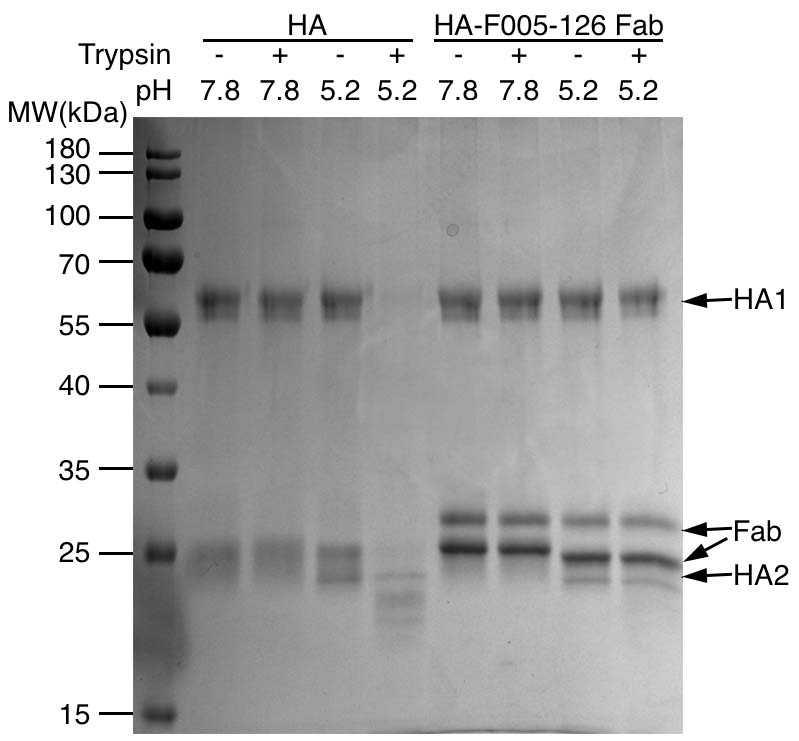

Supplement: S1 Fig — The ectodomain of HA is digested by trypsin after the incubation of 30 minutes under pH 5.2 with or without Fab F005-126. The bound Fab F005-126 prevents the pre-postfusion transition of the HA and renders the HA trypsin resistant. (TIF) [file ppat.1009062.s001.tif]

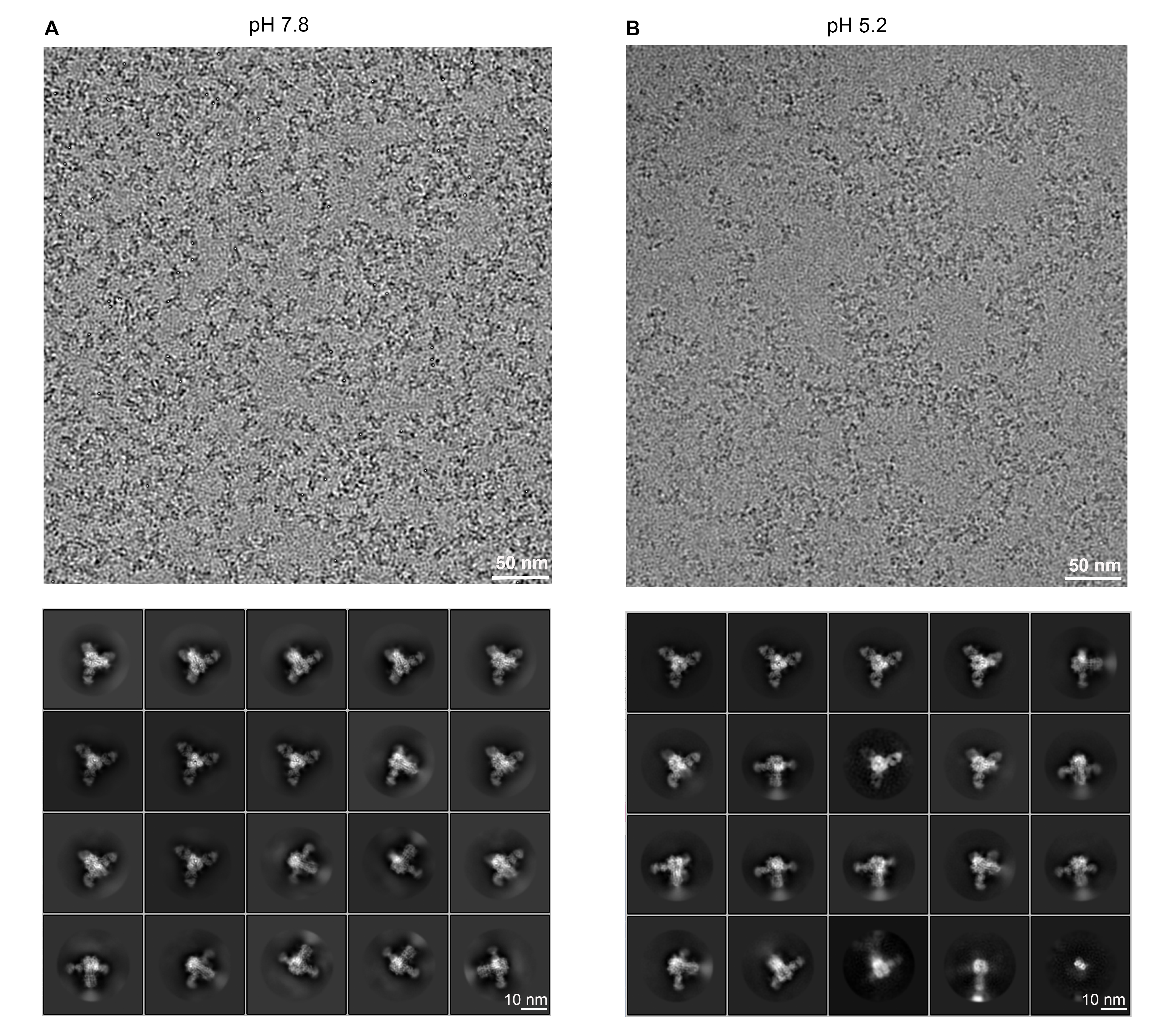

Supplement: S2 Fig — (A) A representative raw micrograph (top) and 2D class averages (bottom) of the HA-Fab complex at pH 7.8. (B) A representative raw micrograph (top) and 2D class averages (bottom) of the HA-Fab complex at pH 5.2. Scale bars in the raw micrographs represent 50 nm. Scale bars in the 2D class averages represent 10 nm. (TIF) [file ppat.1009062.s002.tif]

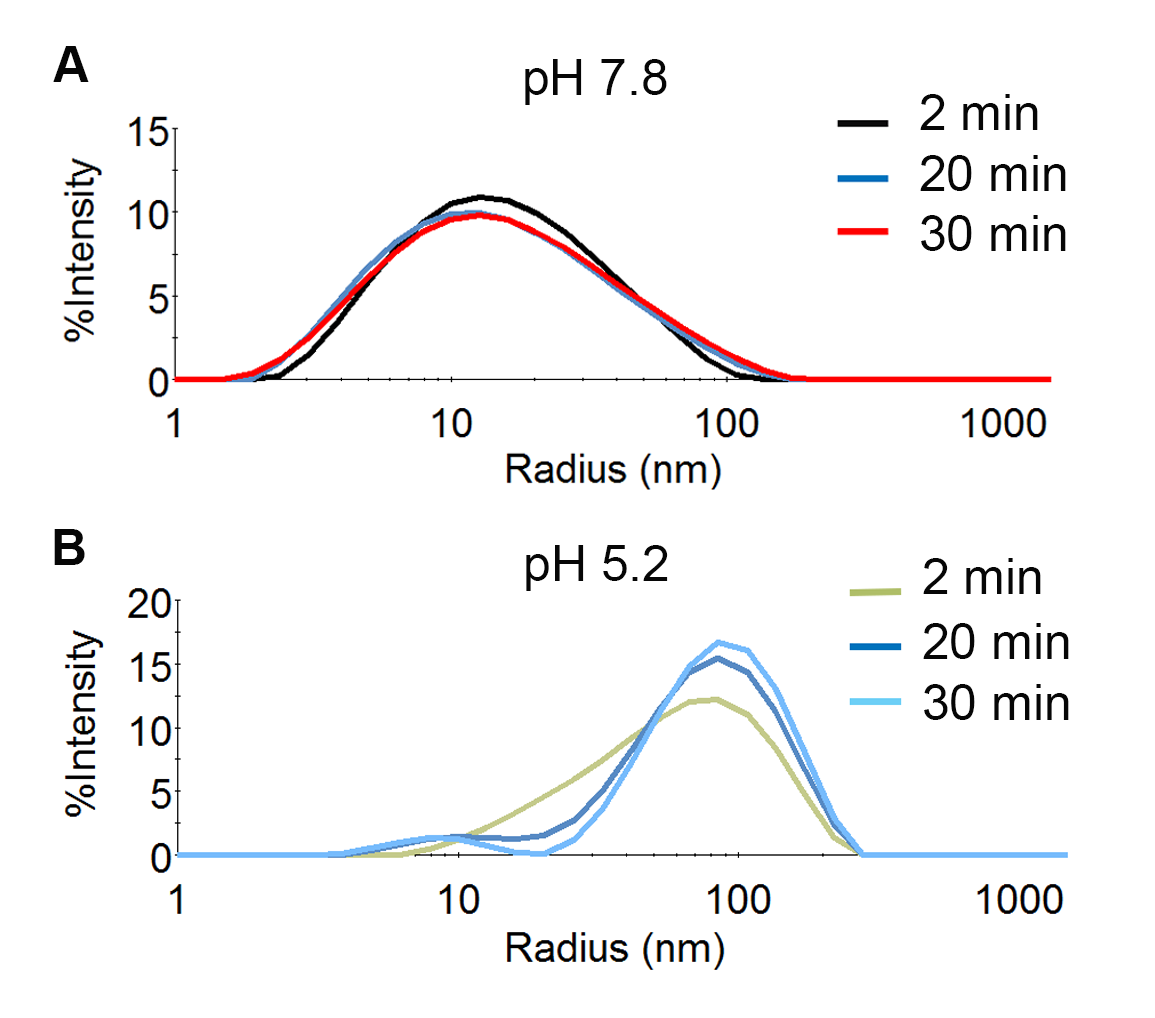

Supplement: S3 Fig — Curves measured at three time points were shown. The neutral pH sample is in a buffer containing 150 mM NaCl, 20 mM HEPES at pH 7.8 and 0.003% LMNG and the complex is at a concentration of 0.07 mg/ml. The low pH sample is in a buffer containing 150 mM NaCl, 20 mM HEPES at pH 5.2 and 0.003% LMNG and the complex is at a concentration of 0.07 mg/ml. (A) Size distribution of the HA-Fab complex at pH 7.8. (B) Size distribution of the low-pH treated HA-Fab complex. (TIF) [file ppat.1009062.s003.tif]

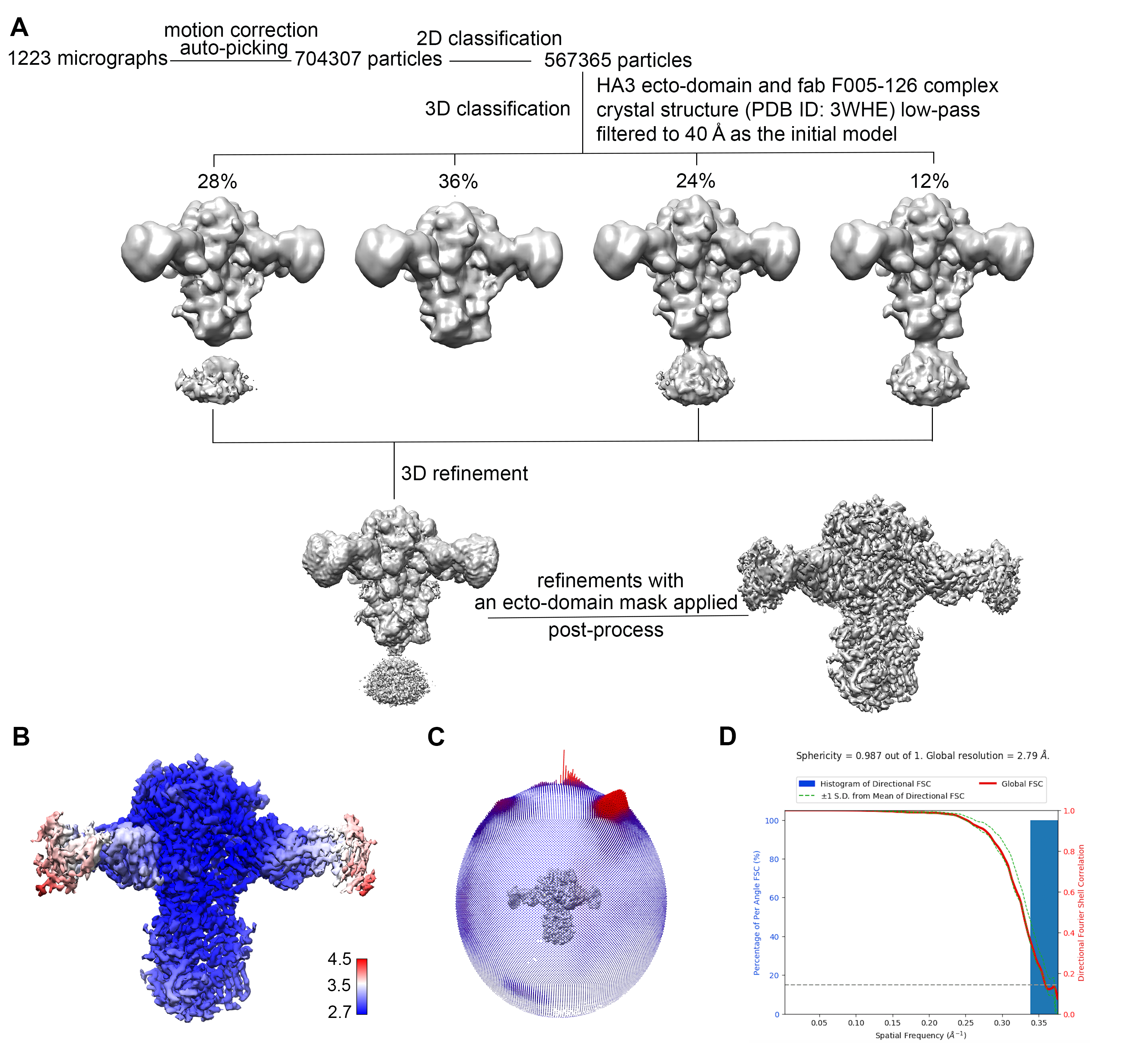

Supplement: S4 Fig — (A) Cryo-EM data processing flowchart. See Materials and methods for details. (B) Local resolution map. (C) Particle orientation distribution. (D) Directional FSC plot for the reconstruction calculated on the 3DFSC server. Sphericity indicates the degree of anisotropy present in the reconstruction. Histogram indicates the portion of voxels with a particular resolution. (TIF) [file ppat.1009062.s004.tif]

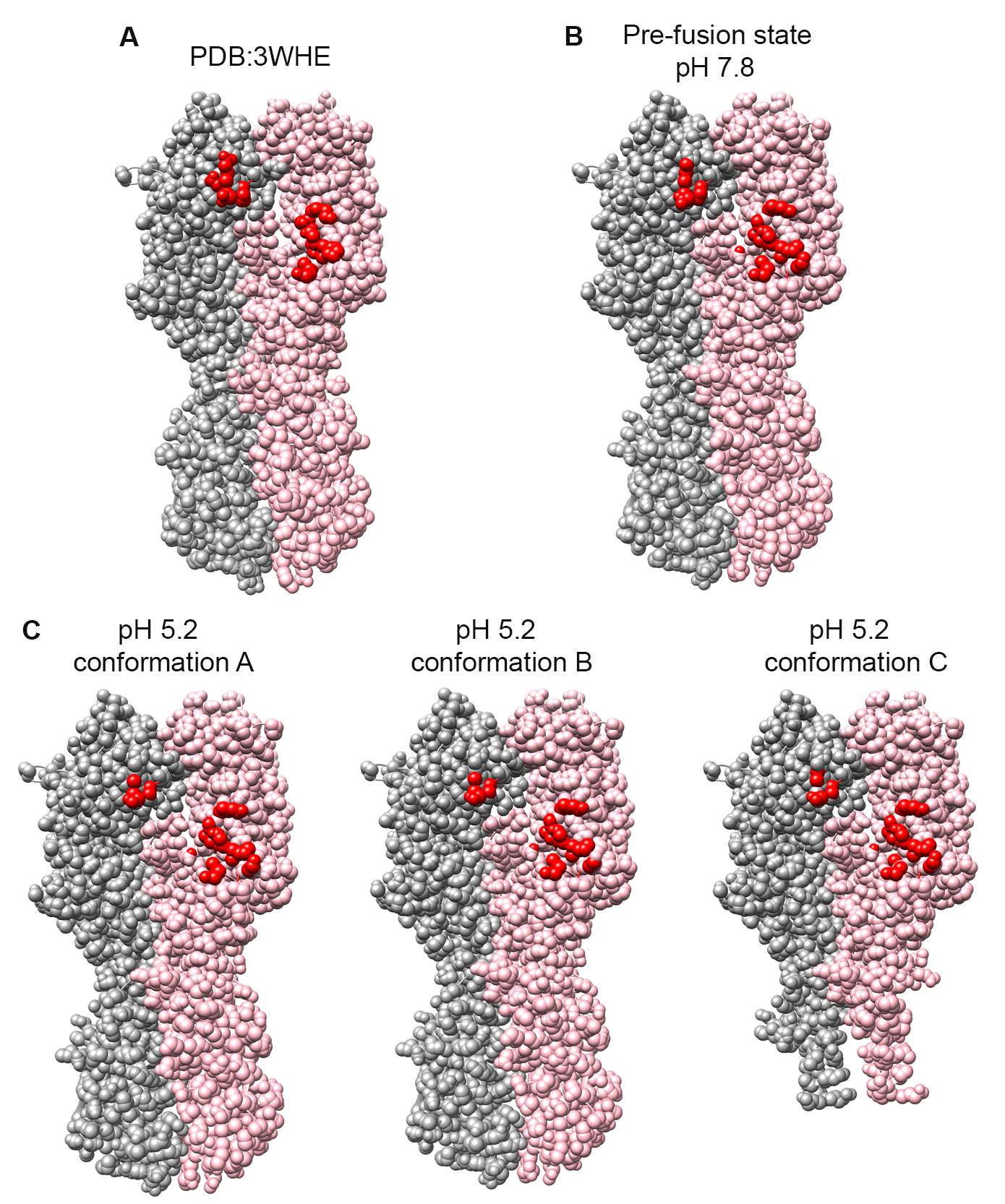

Supplement: S5 Fig — The two HA protomers in the trimer are colored dark grey and light pink color, respectively. (A) The residues involved in direct interactions with Fab F005-126 in the reported crystal structure (PDB accession number: 3WHE) were shown in red color. (B-C) The contacting interface (in red) of HA with Fab F005-126 in different conformations. The contacting interface was calculated with Chimera. Two atoms are considered to have close contacts if the distance between them minus the sum of their van der Waals radii is less than 1. (TIF) [file ppat.1009062.s005.tif]

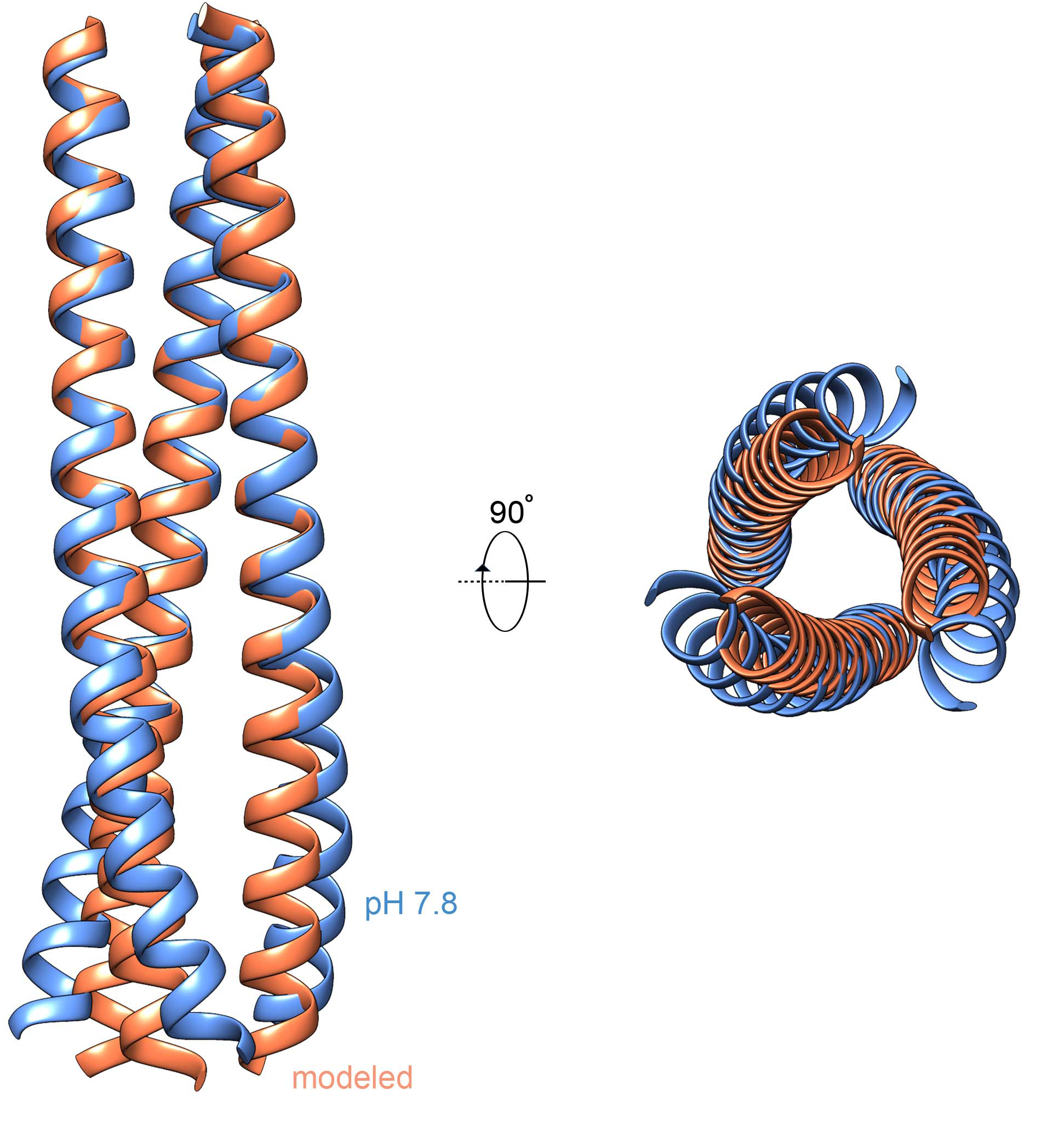

Supplement: S6 Fig — The central helices at pH 7.8 (cornflower blue) superimposed with a model (coral) generated by using the coiled coil parameters of the Helix Cs. Side view (left) and bottom view (right) are shown. The Helix Ds are more open and have a larger twist compared to the simulated coiled coil. The significant structure differences between the simulated coiled coil and the central helices also indicate differences in the coiled coil parameters of the Helix Cs and the Helix Ds. (TIF) [file ppat.1009062.s006.tif]

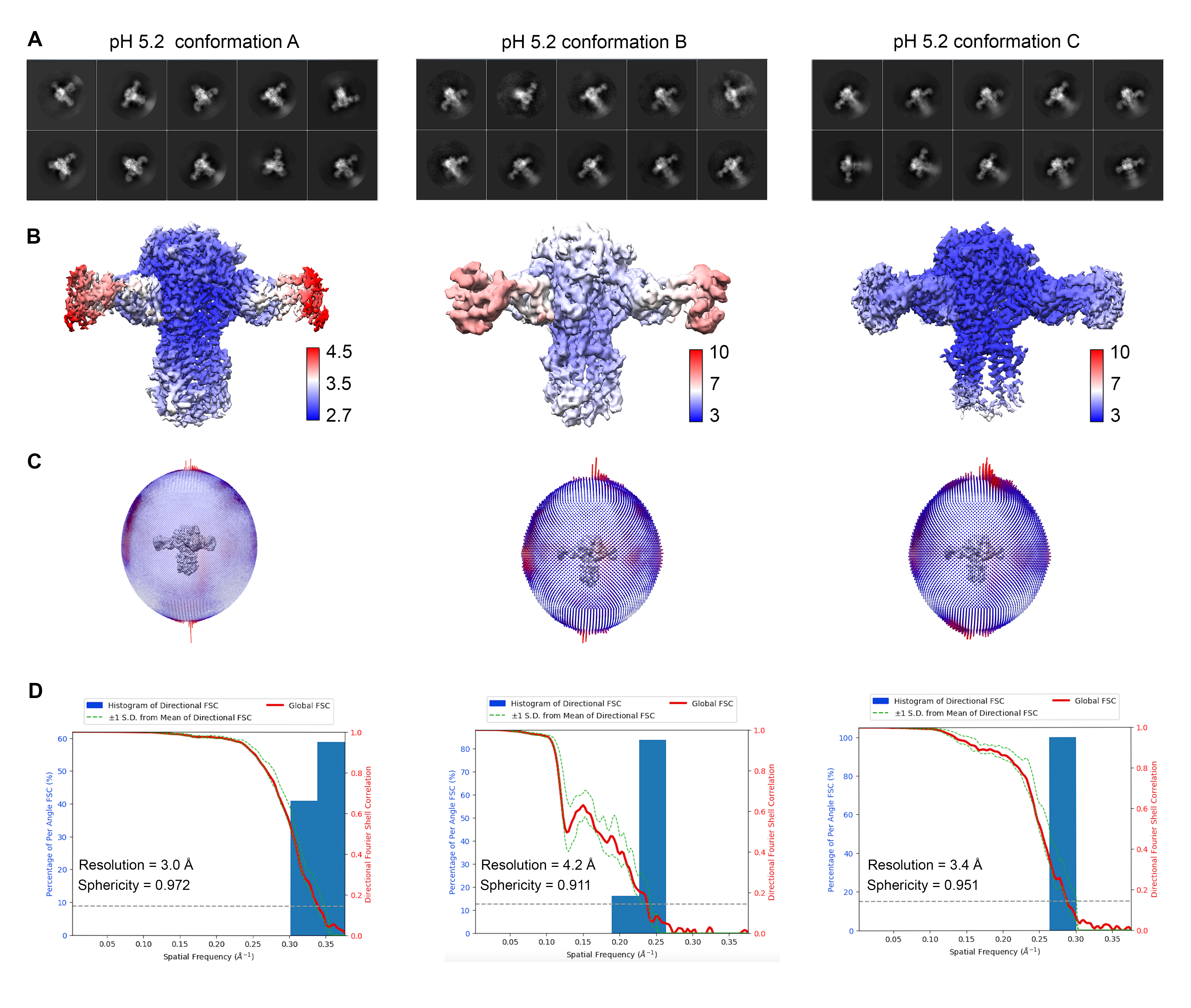

Supplement: S7 Fig — (A-D) Representative 2D class averages (A), local resolution (B), particle orientation distribution (C) and directional FSC plots of the HA-Fab reconstructions at pH 5.2 (D). Left, pH 5.2 conformation A. Middle, pH 5.2 conformation B. Right, pH 5.2 conformation C. The directional FSC plots for the reconstructions are calculated on the 3DFSC server. Sphericities indicate the degree of anisotropy present in the reconstructions. Histograms indicate the portion of voxels with a particular resolution. (TIF) [file ppat.1009062.s007.tif]

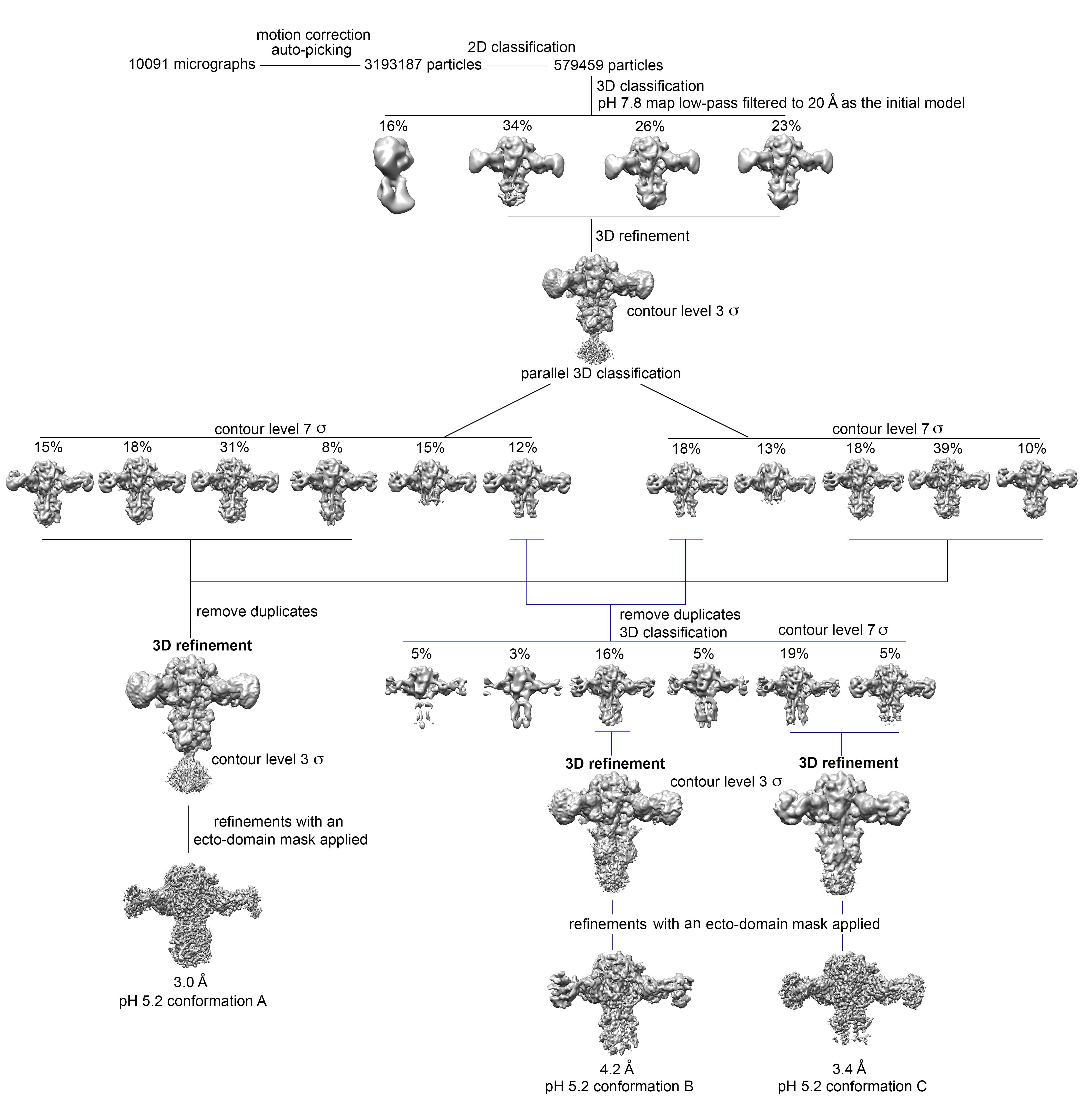

Supplement: S8 Fig — See Materials and methods for details. (TIF) [file ppat.1009062.s008.tif]

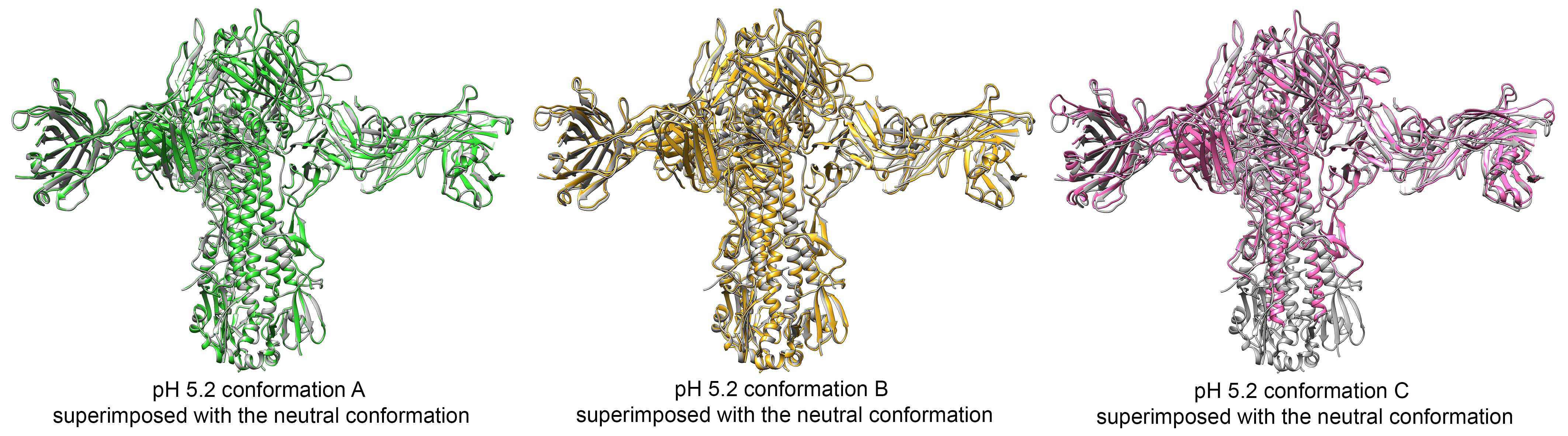

Supplement: S9 Fig — Structure superimpositions of the pH 5.2 conformation A (left, green), pH 5.2 conformation B (middle, gold) and pH 5.2 conformation C (right, hot pink) with the conformation at pH 7.8 (gray), respectively. The r.m.s.d. values between the 813 aligned Cα atom pairs of HA heads (residues 43–313 of HA1) of pH 5.2 conformation A, pH 5.2 conformation B and pH 5.2 conformation C with the conformation at pH 7.8 are 0.25 Å, 0.53 Å and 0.36 Å, respectively. (TIF) [file ppat.1009062.s009.tif]

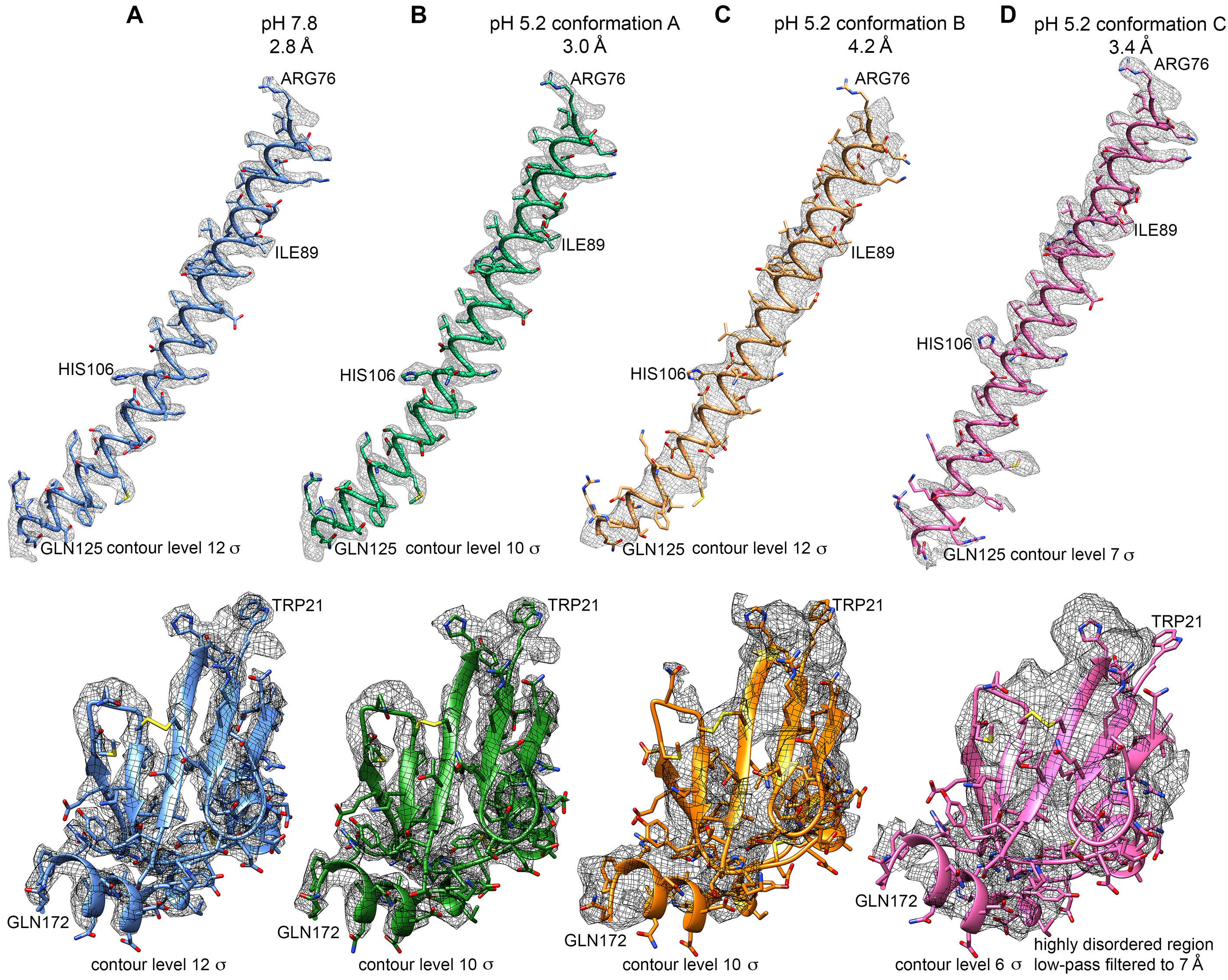

Supplement: S10 Fig — (A-D) Densities around the central helix (residues 76–125 of HA2) and the beta sheet (residues 9–18 of HA1, 21–38 and 126–141 of HA2) in the stem region of different conformations. Density maps are shown as meshes. Residue side chains are shown in balls and sticks with oxygen atoms colored red, nitrogen atoms colored blue and sulfur atoms colored yellow. The contour levels of the maps are listed under each conformation. (TIF) [file ppat.1009062.s010.tif]

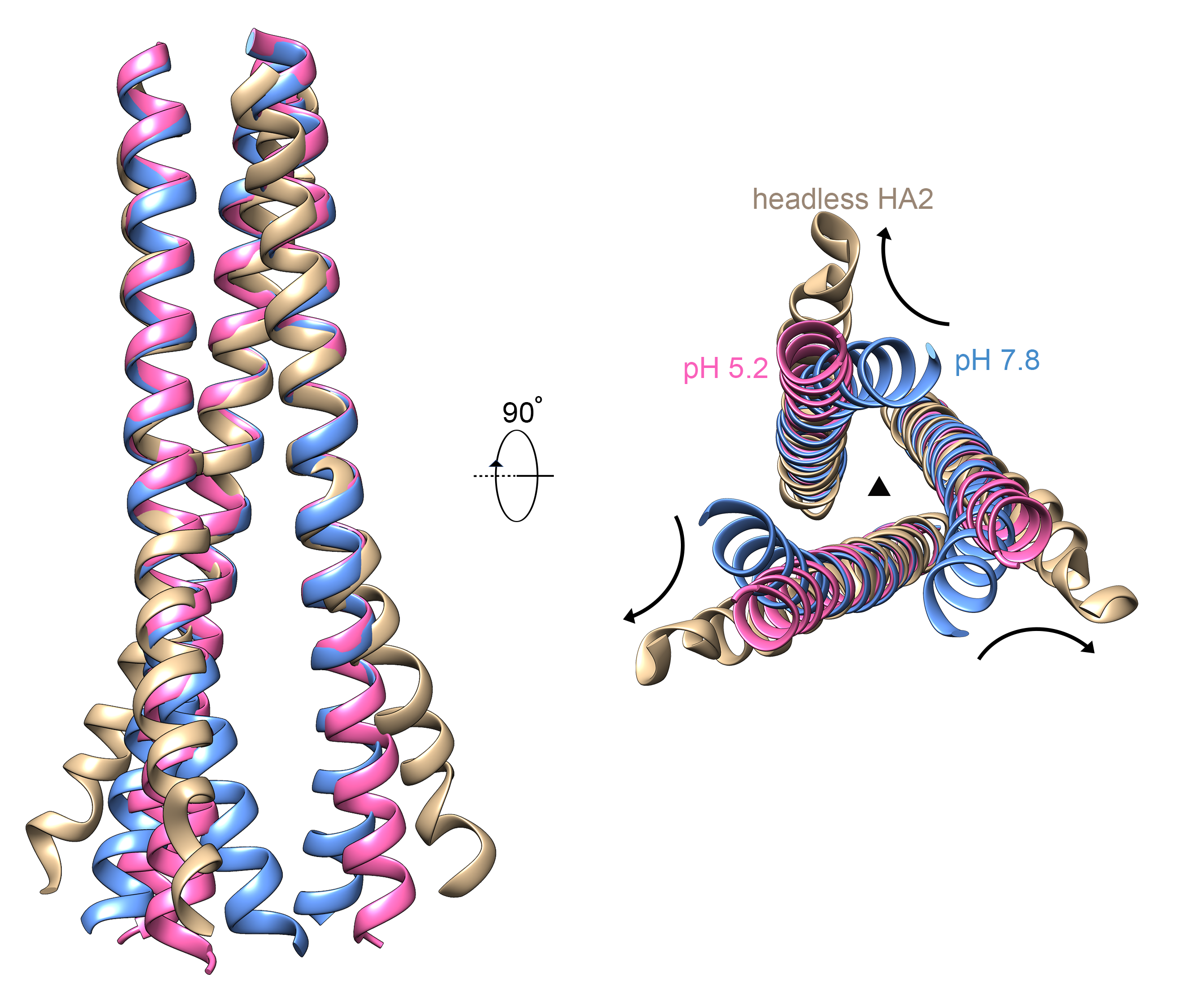

Supplement: S11 Fig — The central helices of the pH 7.8 conformation (cornflower blue), the pH 5.2 conformation C (hot pink) and the headless HA (PDB accession number: 5CJQ), which could represent different stages in the pre-post transition, are compared. Side view (left) and bottom-up view (right) are shown, respectively. Position of three-fold axis in the bottom-up view is indicated by a black triangle. Possible subsequent conformational changes of the Helix Ds in the pre-post transition are indicated by the black arrows. (TIF) [file ppat.1009062.s011.tif]

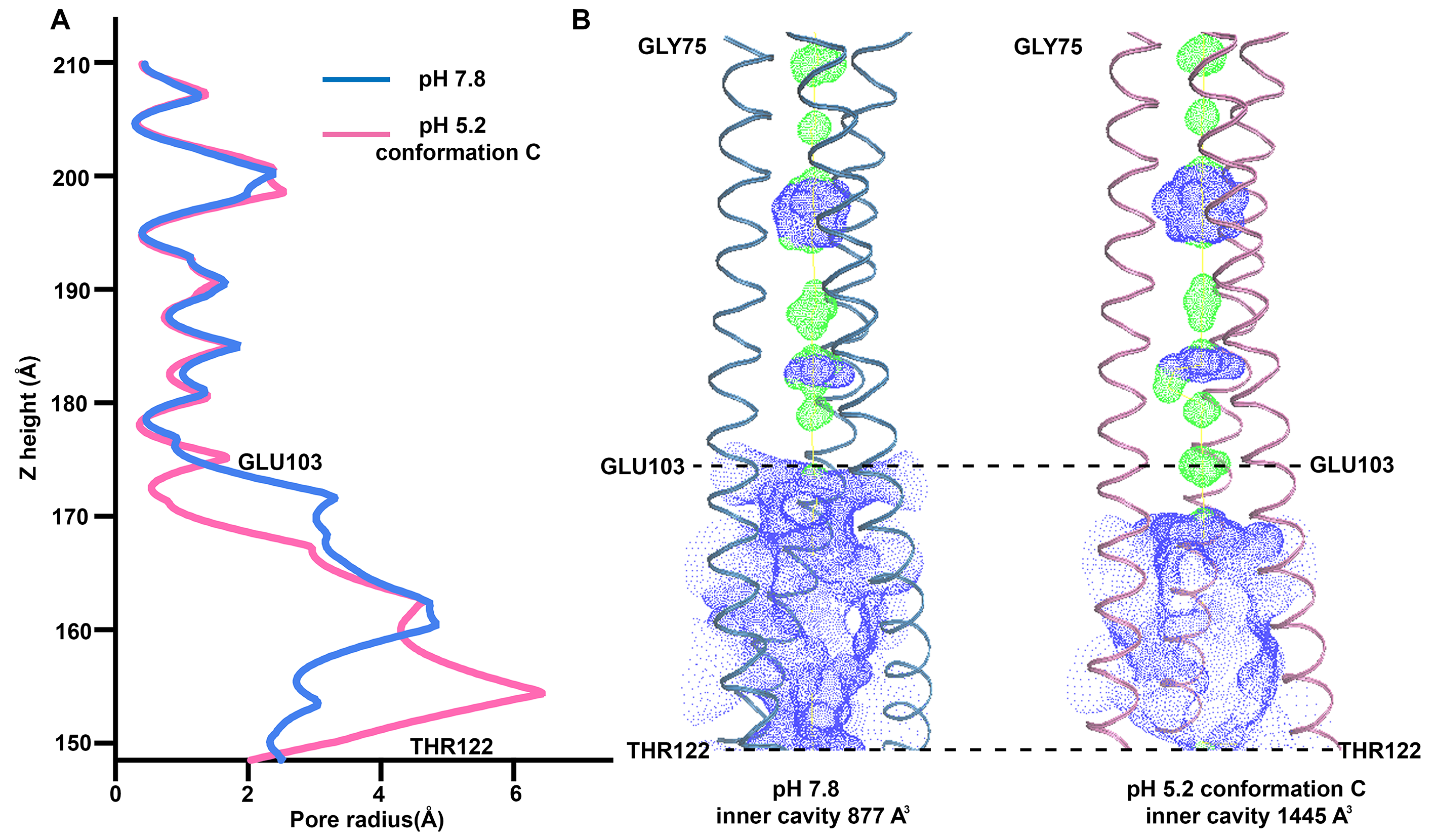

Supplement: S12 Fig — (A) Changes in the pore radius along the Z axis. (B) Comparisons between the inner cavities of the neutral and low pH conformations. Cavities colored green are only enough to adapt single water molecule. Cavities colored blue are large enough to adapt more than two water molecules [53]. (TIF) [file ppat.1009062.s012.tif]

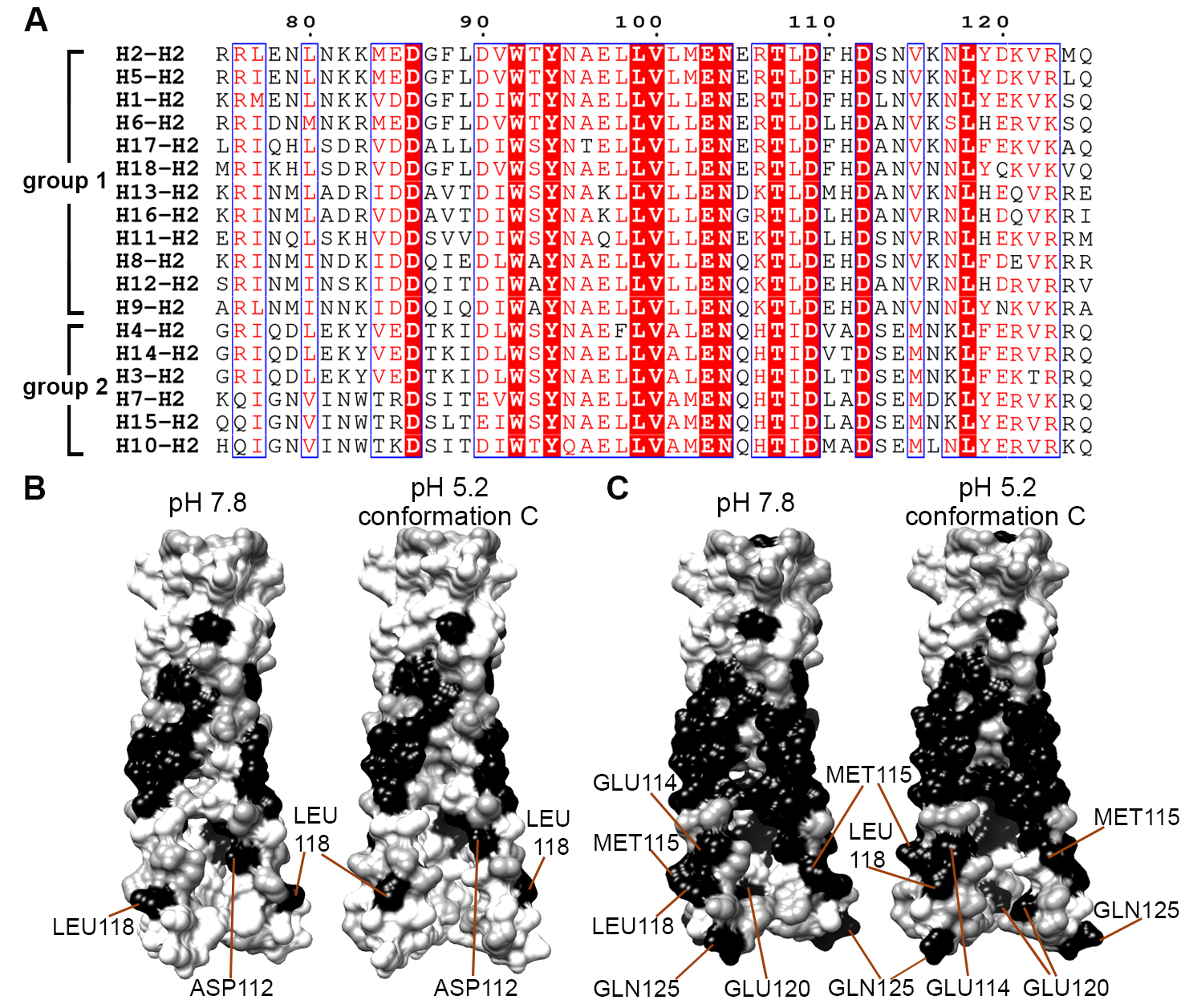

Supplement: S13 Fig — (A) Sequence alignments of the HA2s. The completely conserved residues are shown in white on a red background. The conserved residues are boxed. (B) Surface rendered diagrams showing the pH induced conformational changes of the conserved residues on the surface of the central helices. Residues completely conserved in both the group 1 and group 2 HA2s are colored black. Residues conserved in both group 1 and group 2 HA2s are colored dark gray. Residues not conserved are colored white. (C) Surface rendered diagrams showing the pH induced conformational changes of the conserved residues on the surface of the central helices of the group 2 HA2s. Residues completely conserved in the group 2 HA2s are colored black. Residues conserved in the group 2 HA2s are colored dark gray. Residues not conserved are colored white. (TIF) [file ppat.1009062.s013.tif]

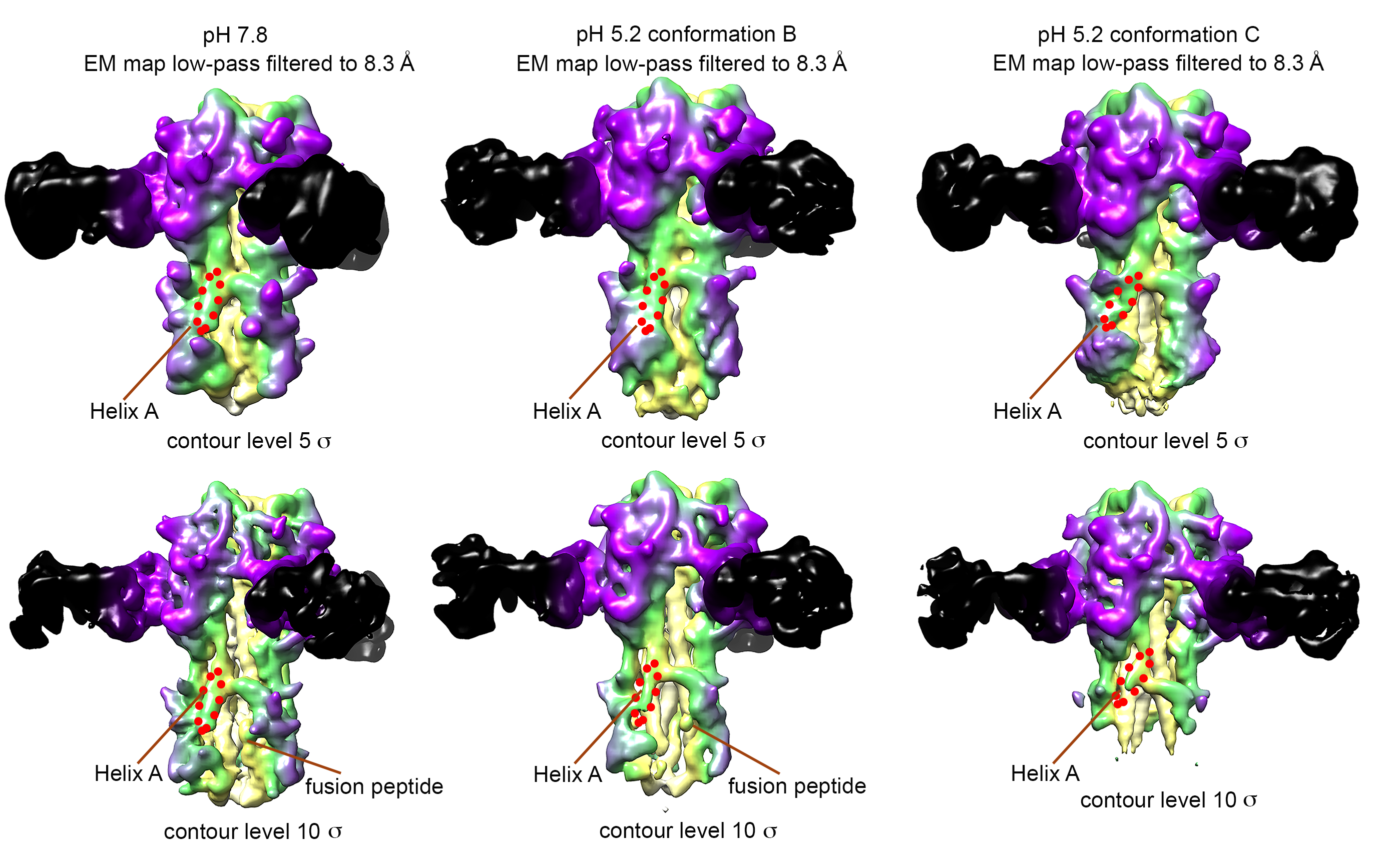

Supplement: S14 Fig — Voxels are colored according to their distances to the three-fold axis. All the maps were low-pass filtered to 8.3 Å. Positions of the Helix As and the fusion peptides are indicated. (TIF) [file ppat.1009062.s014.tif]

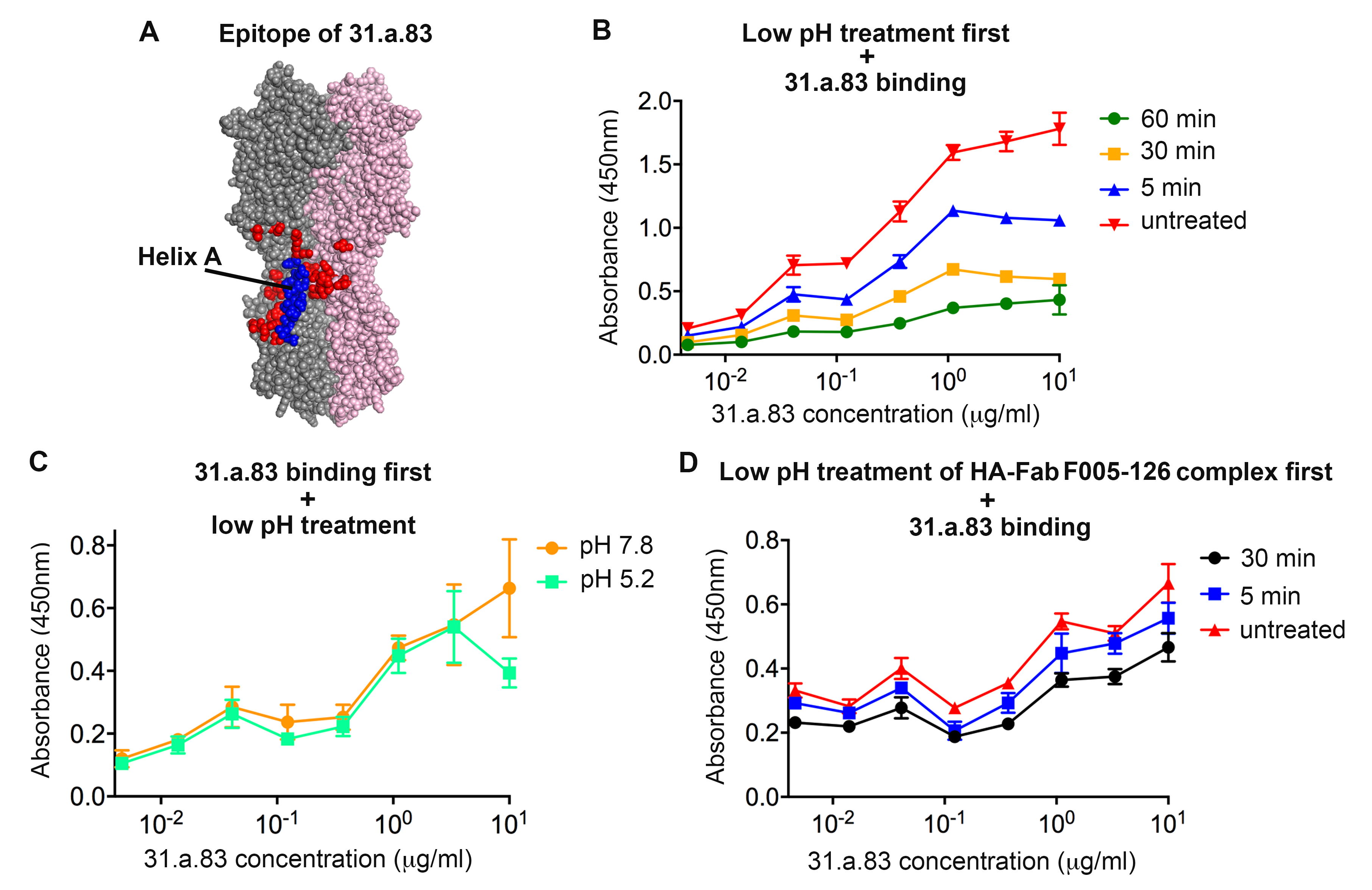

Supplement: S15 Fig — The stem specific antibody 31.a.83 was serially diluted and detected for its binding to HA. Low pH treatments were taken under 37°C. (A) The epitope of 31.a.83 is shown in a space-filling model. The two HA protomers are colored dark grey and light pink, respectively. Residues involved in the epitope of 31.a.83 based on the reported structure (PDB accession number: 5KAQ) are colored red or blue. Residues in blue belong to Helix A. (B) 31.a.83 binding to the low pH treated HA. (C) Serially diluted 31.a.83 was added to the HA sample before low pH treatment and comparisons of 31.a.83 binding to the HA under neutral and low pH conditions by using the premixed samples. (D) 31.a.83 binding to the low pH treated HA-Fab F005-126 complex. All the measurements were repeated three times. (TIF) [file ppat.1009062.s015.tif]
