# Supplementary material for: Structural intermediates in the low pH-induced transition of influenza hemagglutinin
Source: PLoS Pathog. 2020 Nov 30;16(11):e1009062. doi: 10.1371/journal.ppat.1009062 (PMC7728236; doi:10.1371/journal.ppat.1009062)
Supplement: S1 Table — (DOCX) [file ppat.1009062.s016.docx]

**S1 Table. Cryo-EM data collection, image processing and structure refinement statistics.**

|  | HA pH 7.8 | HA pH 5.2 conformation A | HA pH 5.2 conformation B | HA pH 5.2 conformation C |
| --- | --- | --- | --- | --- |
| **Data collection** | | | | |
| Microscope | Titan Krios | Titan Krios | | |
| Detector | K2 Summit | K2 Summit | | |
| Voltage (keV) | 300 | 300 | | |
| Nominal magnification | 105,000× | 105,000× | | |
| Electron exposure (e^-^/Å^2^) | 50 | 50 | | |
| Defocus range set during data acquisition (μm) | -1.0 to -3.5 | -1.0 to -3.5 | | |
| Pixel size (Å) | 1.33 | 1.33 | | |
| **Data Processing** | | | | |
| Final particles | 359,922 | 227,854 | 14,650 | 63,711 |
| Map resolution (Å) | 2.8 | 3.0 | 4.2 | 3.4 |
| Model composition |  |  |  |  |
| Chains | 12 | 12 | 12 | 12 |
| Atoms | 21561 | 21561 | 21426 | 19038 |
| Residues | 2712 | 2712 | 2691 | 2394 |
| Ligands | 6 BMA, 27 NAG, 21 MAN | 6 BMA, 27 NAG, 21 MAN | 6 BMA, 27 NAG, 21 MAN | 6 BMA, 24 NAG, 21 MAN |
| **Refinement and validation** | | | | |
| Resolution limit set in refinement (Å) | 2.8 | 3.0 | 4.2 | 3.4 |
| Correlation coefficient (CCmask) | 0.84 | 0.85 | 0.73 | 0.85 |
| C_ref_ (masked) (Å) | 2.8 | 3.0 | 4.1 | 3.3 |
| Root-mean-square deviation (bond lengths) (Å) | 0.005 | 0.007 | 0.004 | 0.008 |
| Root-mean-square deviation (bond angles) (Å) | 0.645 | 0.693 | 0.731 | 0.67 |
| B factors (Protein) (Å2) | 75 | 75 | 75 | 76 |
| B factors (Ligand) (Å2) | 73 | 73 | 73 | 73 |
| MolProbity Score | 1.81 | 1.83 | 1.97 | 1.79 |
| Clash score | 9.38 | 8.93 | 12.81 | 8.54 |
| Rotamer outliers (%) | 0.13 | 0 | 0.17 | 0 |
| Ramachandran (favored) (%) | 95.47 | 94.83 | 94.94 | 95.2 |
| Ramachandran (outliers) (%) | 0 | 0 | 0 | 0 |
| EMDB ID | EMD-22652 | EMD-22653 | EMD-22654 | EMD-22655 |
| PDB ID | 7K37 | 7K39 | 7K3A | 7K3B |
