# Supplementary material for: Structural intermediates in the low pH-induced transition of influenza hemagglutinin
Source: PLoS Pathog. 2020 Nov 30;16(11):e1009062. doi: 10.1371/journal.ppat.1009062 (PMC7728236; doi:10.1371/journal.ppat.1009062)
Supplement: S2 Table — (DOCX) [file ppat.1009062.s017.docx]

**S2 Table. Model building statistics.**

| Structures | Built segments | | | |
| --- | --- | --- | --- | --- |
|  | HA1^a^ | HA2^b^ | F005-126 Fab Light chain | F005-126 Fab Heavy chain |
| pH 7.8 | 9-326 | 1-172 | 2-26, 34-92, 100-217 | 1-136, 146-221 |
| pH 5.2 conformation A | 9-326 | 1-172 | 2-26, 34-92, 100-217 | 1-136, 146-221 |
| pH 5.2 conformation B | 9-324 | 8-172 | 2-26, 34-92, 100-217 | 1-136, 146-221 |
| pH 5.2 conformation C | 18-324 | 49-125 | 2-26, 34-92, 100-217 | 1-136, 146-221 |

^a^ Residues 1-8 of HA1 are the signal peptide. ^b^ Residues 173-222 of HA2 are the transmembrane domain and the C-terminal cytoplasmic tail.
